# Modeling Linguistic Variables With Regression Models: Addressing Non-Gaussian Distributions, Non-independent Observations, and Non-linear Predictors With Random Effects and Generalized Additive Models for Location, Scale, and Shape

**DOI:** 10.3389/fpsyg.2018.00513

**Published:** 2018-04-16

**Authors:** Christophe Coupé

**Affiliations:** Laboratory Dynamique du Langage, CNRS and University of Lyon, Lyon, France

**Keywords:** mixed-effects models, generalized linear models, generalized additive models, smooth terms, phonemic inventory size, Delaporte distribution, Box-Cox *t* distribution, GAMLSS

## Abstract

As statistical approaches are getting increasingly used in linguistics, attention must be paid to the choice of methods and algorithms used. This is especially true since they require assumptions to be satisfied to provide valid results, and because scientific articles still often fall short of reporting whether such assumptions are met. Progress is being, however, made in various directions, one of them being the introduction of techniques able to model data that cannot be properly analyzed with simpler linear regression models. We report recent advances in statistical modeling in linguistics. We first describe *linear mixed-effects regression models* (LMM), which address grouping of observations, and *generalized linear mixed-effects models* (GLMM), which offer a family of distributions for the dependent variable. *Generalized additive models* (GAM) are then introduced, which allow modeling non-linear parametric or non-parametric relationships between the dependent variable and the predictors. We then highlight the possibilities offered by *generalized additive models for location, scale, and shape* (GAMLSS). We explain how they make it possible to go beyond common distributions, such as Gaussian or Poisson, and offer the appropriate inferential framework to account for ‘difficult’ variables such as count data with strong overdispersion. We also demonstrate how they offer interesting perspectives on data when not only the mean of the dependent variable is modeled, but also its variance, skewness, and kurtosis. As an illustration, the case of phonemic inventory size is analyzed throughout the article. For over 1,500 languages, we consider as predictors the number of speakers, the distance from Africa, an estimation of the intensity of language contact, and linguistic relationships. We discuss the use of random effects to account for genealogical relationships, the choice of appropriate distributions to model count data, and non-linear relationships. Relying on GAMLSS, we assess a range of candidate distributions, including the Sichel, Delaporte, Box-Cox Green and Cole, and Box-Cox *t* distributions. We find that the Box-Cox *t* distribution, with appropriate modeling of its parameters, best fits the conditional distribution of phonemic inventory size. We finally discuss the specificities of phoneme counts, weak effects, and how GAMLSS should be considered for other linguistic variables.

## The Growing Weight of Statistics in Linguistics

Different reasons can be put forward for why data-driven approaches are gaining more prominence in the whole linguistic field. First, large digital datasets such as *WALS* (Dryer and Haspelmath, 2013), *ASJP* (Wichmann et al., 2016), *Lapsyd* (Maddieson et al., 2013), or *D-Place* (Kirby et al., 2016) are freely and readily available for computational analysis. Second, personal computers now offer high computational power, along with efficient and open-source statistical software, like the *R language and environment for statistical computing and graphics* (R Development Core Team, 2017). In particular, advanced modeling techniques that were either still under development or computationally out of reach with affordable computers two decades ago are becoming accessible. Third, such techniques are exported from fields such as econometrics, ecology or genetics to linguistics. While the trend of ‘big data’ is already well established in subfields of linguistics such as text mining, it has also more recently gained prominence in studies of language diversity or language change. It is for example becoming increasingly common to publish studies investigating more than a thousand languages (e.g., Wichmann et al., 2011; Moran et al., 2012). This is true in particular when the relevance of non-linguistic factors such as sociodemographic ones is being investigated.

With these approaches comes a number of issues regarding the choice of appropriate statistical modeling for the questions at stake. The illusion of truth is dangerous, especially when algorithms deliver arrays of *p*-values without warning of possible misspecifications or violated assumptions. Such issues are a component of the crisis of confidence in psychology (e.g., Earp and Trafimow, 2015): widespread failure to replicate previous studies may be due to different factors, but one of them is likely the inappropriate use of statistical models (e.g., Greenland et al., 2016). This is compounded by the fact that articles often do not report whether the authors have properly checked the assumptions of their tests, nor give sufficient information to replicate the experiment.

## A Case Study: the Size of Phonemic Inventories

What drives linguistic diversity? What phenomena, and in particular what external factors, explain the distribution of linguistic structures across the globe? These questions are at the heart of linguistics, and can be considered at various levels of linguistic analysis, either with qualitative or more quantitative approaches. At the phonological level, one of these approaches consists in studying phonemic inventories, and how their size varies across linguistic families and areas. Phonemic inventory size has thus been tentatively related to two non-linguistic variables, namely population size (Hay and Bauer, 2007) and the distance from Africa (Atkinson, 2011a,b), with reference in the second case to modern humans’ migrations out of this continent during the last 100,000 years. Both proposals have led to substantial debates (e.g., Pericliev, 2004; Bybee, 2011; Donohue and Nichols, 2011; Moran et al., 2012), both at a theoretical and at a methodological level. Beyond that, language contact and subsequent borrowing – or lack of it –, but also inheritance from parent languages, are obvious partial answers to why a phonemic inventory may be small or large.

In the next sections, we perform as series of regression analyses of phonemic inventory size, in order to illustrate the potentialities and limits of various approaches. In order to do this, we built a dataset of 1529 languages containing 681 languages from the *Lapsyd* database (Maddieson et al., 2013), complemented by 846 languages from the *Phoible* database (Moran et al., 2014). This dataset compiles information for a number of predictors:

- Linguistic families and number of speakers extracted from the *WLMS* dataset (Global Mapping International SIL International, 2012);
- Distance from Africa computed following Atkinson’s methodology with a departure point located in eastern Africa (36°W, 8°N) and great circle distances constrained by specific passage points (Sinai region, Bering Strait etc.) (Atkinson, 2011b);
- A measure of local linguistic density, equal for each language to the number of languages spoken less than 50 km away, on the basis of the polygons delimiting the respective areas of these languages, again given by the WLMS dataset; computations were performed with QGIS (Quantum GIS Development Team, 2017).

There were 139 linguistic families, including a number of families restricted to a single language in the case of isolates or creoles. Several transformations were applied to the continuous variables: (i) a natural logarithm transformation was applied to the number of speakers, (ii) a cubic root transformation was applied to the local linguistic density, since it allows to expand the range of values without the issues raised by the log transformation, especially with 0 values, (iii) a scaling without centering was applied to all continuous variables – which is to say, we divided the values of each variable by the standard deviation of this variable, in order to be able to compare their respective effect sizes in the models.

The choice of predictors reflects recent proposals in the relevant literature, and includes heavily debated variables such as Atkinson’s distance from Africa. Together, these predictors provide a rich testbed for the various models considered hereafter; conversely, these models may shed new light on current issues in linguistic diversity, at least at a statistical level with better modeling of the putative influence of geographic and social factors.

**FIGURE 1:**
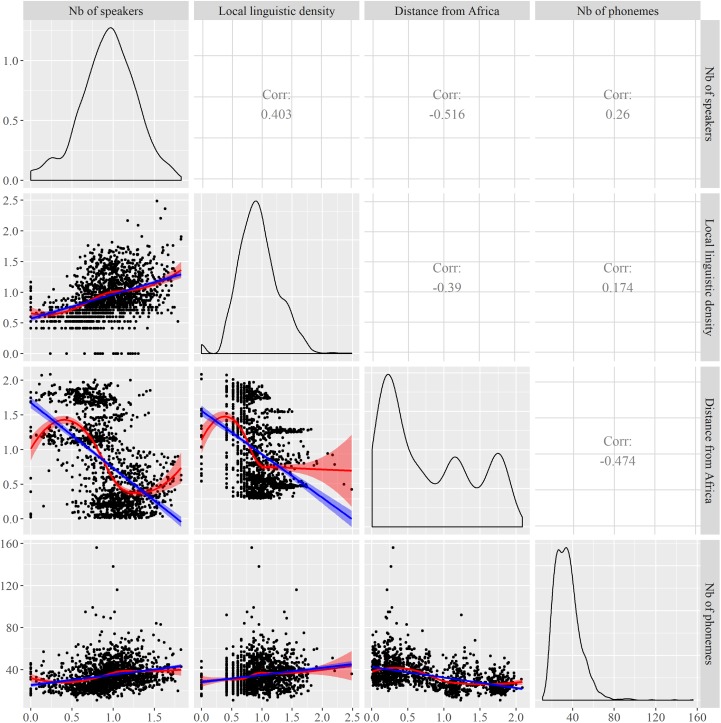
Relationships between *Number of phonemes*, *Number of speakers*, *Distance from Africa*, and *Local language density*. The curves on the diagonal are density curves. The blue lines are linear regressions, the red curves loess regressions.

**Figure 1** provides an overview of *Number of Speakers, Local linguistic density, Distance from Africa*, and *Phonemic inventory size*, and of their one-to-one relationships. The density function of *Distance from Africa* is noticeable because of its three components. These components are related to the distribution of languages on the planet according to the distance from the reference point in eastern Africa. The leftmost component encompasses languages from families such as the Nilo-Saharan, Niger-Congo, and Afro-Asiatic families (left side of the component), but also the Indo-European, Dravidian, Sino-Tibetan, and Austro-Asiatic families (right side of the component). The second component relates mostly to the Trans-New-Guinean, Australian, and Austronesian families, while the rightmost component relates to languages spoken in the Americas, such as Tupi, Macro-Ge, or Arawakan languages. With respect to the locations of passage points for the computation of distances, the “bumps” arise by contrast with regions of lower linguistic diversity, such as in western central Asia and at high latitudes, e.g., in the region of the Bering Strait.

All the regression models were built within the R environment (version 3.4.3) (R Development Core Team, 2017), using various packages that are cited in the following sections. The code used to produce the results and the figures can be found in Supplementary Presentation 1. We remain in a frequentist framework, and therefore do not refer to packages offering Bayesian approaches. Our models always include *Distance from Africa*, *Number of Speakers* and *Local linguistic density* as fixed continuous effects, and *Family* as an intercept random effect for reasons given in section 3.1. The dependent/predicted variable is always *Phonemic inventory size*, also called *Number of phonemes*. In summaries of models, *p*-values lower than 0.05 are in bold, but all exact *p*-values are given unless very small – smaller than 0.001.

## Advances in Statistical Modeling in Linguistics

How can one identify relationships between phonemic inventory size and the set of predictors mentioned above? Regression models are one of the main methodological answers, especially since they account for several predictors simultaneously. Indeed, the one-to-one relationships between the dependent variable and the various predictors, as exemplified in **Figure 1**, must be considered in the light of a possibly complex network of dependencies between the latter. Since the absence of strong multicollinearity is a prerequisite of regression models, we checked it by computing the variance inflation factors, or VIF, of the continuous predictors. The three values are between 1 and 1.5, which allow safely concluding to low multicollinearity – values higher than 4 or 5 would have been problematic.

### From Linear Regression Models to Mixed-Effects Linear Regression Models

As said previously, regression models relating a *dependent variable*, also known as a *response variable*, to a number of *predictors*, also known as *independent variables* or *explanatory variables*, are common tools. Different approaches, however, fall into this broad category, from straight linear regression to quantile (Cook and Manning, 2013) or ridge (de Vlaming and Groenen, 2015) regression.

There is a growing use of *linear mixed-effects models* (LMM) in linguistics (Jaeger et al., 2011; Johnson, 2014; Winter and Wieling, 2016). In these models, random effects are considered in addition to fixed effects to better account for the distribution of the dependent variable. Random effects allow in particular to account for the issue of non-independence of observations characterized by grouping, known as Galton’s problem, which can lead to what is known as pseudo-replication and therefore to increased type I errors, i.e., erroneous significant results (Hurlbert, 1984). As an example in biology, closely related species are assumed to have more similar traits because of their shared ancestry and hence produce more similar residuals from the least squares regression line. Comparatively, in studies investigating a linguistic phenomenon in a large number of languages, not accounting for the increased likelihood that languages sharing a common ancestor share similar features may lead to wrong conclusions in favor of spurious results. If regression is used, this usually leads to the inclusion of linguistic family as a random effect (Atkinson, 2011b). A strategy used by linguists in the field of typology has also been to avoid non-independence by relying on sampling strategies. In Maddieson (1984, p. 158–159)’s work on phonological inventories, the genetic bias was for example controlled by the following method: “*include no pair of languages which had not developed within their own independent speech communities for at least some 1000–1500 years, but to include one language from within each group of languages which shared a history closer than that.*”. In experimental linguistics, repeated measurements within subjects or within items are also now usually accounted for with random effects (Baayen et al., 2008).

At the statistical level, including random effects is a more reliable strategy than for example averaging values over subjects or items (Baayen et al., 2008). Such a strategy to bypass the independence problem indeed leads to reduced datasets and a significant loss of information. Random effects fall into random intercepts and random slopes, and with the latter, the impact of predictors entered as fixed effects can be further analyzed across groupings of observations (Barr, 2013). More generally, as underlined by Drager and Hay (2012), random effects are not only a tool to get more accurate models; actually looking at the conditional modes of their levels can provide useful information. For example, if the distribution of levels of a subject random effect reveals that lower values are mostly those of males, and higher values mostly those of females, it is very likely that sex should be added as a covariate to the model. Upon doing so, the distribution of levels of the subject random effect will likely no longer display a structure according to sex, and its variance will likely be lower.

As in more complex models presented later in this article, the parameters of a LMM can be estimated with different techniques. Besides Bayesian approaches that we do not cover in this article, *maximum likelihood estimation* or MLE is a commonly employed technique. The underlying algorithms aim at finding values of the parameters which maximize the likelihood of observing the sample of data fed to the model. The higher the likelihood, the better the fit to the data. Usually, the logarithm of the likelihood is given as a measure of the quality of the fit. The so called *log-likelihood* is always negative, and the closer it is to 0, the better the model’s goodness of fit is. Conversely, the deviance, *D*, which is equal to minus twice the natural logarithm of the likelihood, is always positive; again, the closer it is to 0, the better the fit of the model.

Both log-likelihood and deviance are good indicators of the quality of the fit, but one is also often interested in the parsimony of computed models. Reaching a good fit with a high number of parameters is for example less parsimonious than reaching the same fit, or a slightly worse one, with only half of them. The *Akaike Information Criterion* or AIC is commonly used to evaluate parsimony, and penalizes the deviance by twice the number of degrees of freedom in the model, *df*. More precisely, *AIC* = 2.*df* + *D*, and the lower the value, the more parsimonious the model. The factor 2 corresponds to a specific tradeoff, and other criteria rest on other values. The *Bayesian Information Criterion* (BIC), also known as *Schwarz Bayesian Criterion* (SBC), is equal to ln(*n*).*df* + *D*, where *n* is the number of observations, i.e., the sample size. The previous definition of BIC, however, assumes that observations are independent, which is not true for example when data are recorded longitudinally, since there is temporal auto-correlation. In such situations, an ‘effective sample size’ *n′* must replace *n* (Jones, 2011). Compared to the AIC, the BIC more strongly penalizes models with more parameters, and model selection based on it will therefore tend to promote simpler models. The BIC is thus more conservative against overfitting. The number of degrees of freedom which is part of the computation of AIC and BIC is not easy to estimate when random effects are included in the model – one must rely on approximations such as Satterthwaite or Kenward-Roger. Both the AIC and BIC are specific instances of generalized AIC, or GAIC, which is equal to *k*.*df* + *D*, where *k* is a positive real number. There is no a priori reason to choose a specific value of *k* over another, and several measures like AIC and BIC can be used simultaneously to assess the parsimony of several models (Kuha, 2004). Information criteria are hence useful when one tries to select the most appropriate model for a given set of observations and possible predictors (Burnham et al., 2011). While there is no significance test associated with AIC or BIC, they offer more flexibility than for example likelihood ratio tests, which require to compare two models that one is nested into the other. The AIC and BIC values reported for the various models in the next sections have all been rounded up or down to the closest whole number. Two identically reported values may therefore be in fact slightly different.

Turning our attention to our test case, we can compute a LMM with the *lmer()* function provided in the lme4 package (Bates et al., 2015) – one of the better-known packages offering this possibility. *lmer()* takes as inputs the dataset and a formula specifying the predicted variable, the fixed effects and the random effects of the desired model, and outputs estimates for the various parameters of this model. The underlying algorithm uses either a maximum likelihood (ML) or a restricted/residual maximum likelihood (REML) approach. The second differs from the first in the way the variance components that belong to random effects are estimated: REML accounts for the loss in degrees of freedom corresponding to fixed effects, while ML does not. While the variances of random effects may be more accurate when REML is used, ML is the only correct approach when comparing models with different fixed effects. In our case, *Distance from Africa*, *Number of speakers*, and *Local linguistic density* are entered as fixed effects, and could not qualify as random effects given their non-categorical nature. Linguistic families (*Family*) are entered as random intercepts, since following Bolker (2015), these families are chosen from the set of all linguistic families, and we are not primarily interested in the differences, in terms of number of segments, between families – we only wish to account for the dependencies the latter create in the data.

A random intercept for a categorical variable with *N* levels additionally requires only one parameter to be estimated – the variance, since the mean is fixed to 0 – while a fixed effect would request *N*-1 parameters. This is true if no random slope is simultaneously considered, since covariance between the random slope and the random intercept must then be estimated unless it has been constrained to take a 0 value. We did not consider random slopes in our models, both for the sake of simplicity and because we hypothesized that the impact of the fixed effects did not vary across the linguistic families. We are aware though that this choice could be contested (Barr, 2013).

**Table 1** summarizes the output of the model. The lmerTest package is loaded so that the *lmer()* function returns *p*-values with Wald *t*-tests. There are two options to approximate the used degrees of freedom: the Satterthwaite approximation, and the Kenward–Roger approximation which is a slightly more conservative option. Likelihood-ratio tests (LRT), which compare the likelihood of the initial model with that of a model where a target fixed parameter has been dropped, are another option to assess significance. Keeping things simple with *t*-tests, the only *p*-value (well) below 0.05 is for the estimate of *Distance from Africa*. It appears that the further away from the reference point in Africa, the smaller the phonemic inventory size. The estimates for the two other fixed predictors are not significantly different from 0.

**Table 1:** Output of a LMM applied to the data.

Predictors	Dependent variable			
	
	Number of phonemes			
	
	Estimate	Standard error	*t*-value	*p*-value
**Fixed parts**				
(Intercept)	37.75	2.46	15.37	**<0.001**
Distance from Africa	-5.44	1.51	-3.61	**<0.001**
Number of speakers	-1.00	1.06	-0.94	0.348
Local linguistic density	1.39	0.97	1.42	0.155

**Random parts**				
σ^2^	91.46			
τ_00,Family_	77.81			
*N*_Family_	139			
ICC_Family_	0.46			
Observations	1,529			
*R*^2^/Ω_0_^2^	0.481/0.478			
AIC	11,435			
Deviance	11,423			


**P-values lower than 0.05 are in bold*.*

**FIGURE 2:**
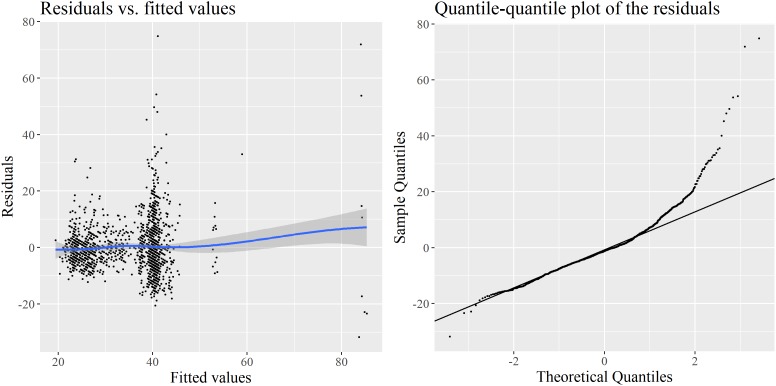
Diagnostics for the LMM model: raw residuals vs. fitted values **(left)** and quantile-quantile plot of these residuals **(right)**.

How much confidence should we put in these results? Their validity rests upon the satisfaction of a number of assumptions (Zuur et al., 2010), among them the normality of the residuals and their constant variance along the fitted values (homoscedasticity). In **Figure 2**, two diagnostic plots reveal that these requirements are not met: there is strong heteroscedasticity of the residuals, and a visually clear departure from normality observable in the quantile-quantile plot. The conclusions from the model should therefore be reported with caution, even if LMM are robust to a certain degree of non-normality.

In order to resolve issues of non-normality of the residuals, one commonly found strategy is to transform the dependent variable, whether it is log-transforming count data or taking the inverse of reaction times. The problem is then, however, that a predictor appearing to be significant with respect to the transformed variable is not necessarily significant with respect to the untransformed one, since the mapping between the transformed and untransformed variables is non-linear. In some cases, hypotheses and underlying processes may well concern the transformed variable and not the raw one, in which case it makes perfect sense to apply a transformation. If this is not the case, models based on a transformed dependent variable may not be very informative. All in all, applying non-linear transformations to the predicted variable as the default strategy to overcome statistical issues is therefore not recommended, although these transformations should not be completely discarded. With respect to count data, a number of articles have been published in ecology to discuss log transformation, and overall favor not transforming the data, although linear models with a log transformation often seem robust with large datasets, and may be more resistant to false positives, also known as type I errors (O’Hara and Kotze, 2010; Ives, 2015; Warton et al., 2016). Looking beyond the frequentist framework, Bayesian approaches to predictive uncertainty allow construction of credible intervals in untransformed units from a regression model with a transformed dependent variable (Gelman and Hill, 2007; Korner-Nievergelt et al., 2015). Within the frequentist framework, other modeling options are available, and are described in the next sections. Given the inadequacy of the previous LMM with respect to our test case, it makes sense to consider such options.

It is worth noting that these issues have been highlighted by some authors with respect to phonemic inventory size: an extract of the supporting online material of Cysouw et al. (2012)’s comment on Atkinson (2011b) mentions that ‘*It has repeatedly been observed that there is a positive correlation between the phoneme inventory size of a language and the speaker community size (S17-S19) (…) Note that for this correlation, we used the logarithm of population size and the logarithm of the phoneme inventory size. The analysis of the expected distribution of phoneme inventory size is still not settled (S20–S22), but using a logarithm seems to be preferable to using the raw numbers*’ (p. 14-15). In Atkinson’s study, rather than raw or log-transformed inventory sizes, an index of complexity of the phonemic inventories, including tones and with a limited range of values, was considered. The distribution of the dependent variable was therefore very different from ours, and we can argue that the raw number of phonemes provides more information than an index of complexity derived from it.

For the sake of exhaustiveness, we considered a model with the logarithm of the number of phonemes as dependent variable. Despite the transformation, the residuals are still rather unsatisfactory, although more homoscedastic and closer to normality than those of the model with untransformed numbers of phonemes. One could here argue that the log transformation is not the most appropriate, and that other approaches could be considered, such as Box-Cox transformations (Box and Cox, 1964).

In cases where relations between observations can be described with tree-like structures, phylogenetic regression methods can be used to appropriately model the expected structure of covariance between observations, and thus prevent autocorrelation (Symonds and Blomberg, 2014). These models are commonly used in biology and take advantage of the phylogenetic trees derived from molecular data. However, as for linguistic data, especially when comparing large numbers of languages from distant families, the degree of confidence in the reconstructed tree is often low, at least in the higher branches. This perhaps explains why many studies rely on family level groups in mixed-effects models, despite this being only a very partial account of the relationships between languages. A slight improvement resides in considering several levels of classification, for example with subfamilies nested within families, but again this is only a partial account of the expected covariance between languages.

### Generalized Linear Mixed-Effects Regression Models

Generalized linear models (GLM), either with or without random effects, are also on the rise. As their name suggest, they extend linear models, in that they allow the dependent variable to follow a distribution other than Gaussian (the Gaussian distribution which is also called normal distribution). They are particularly, but not only, useful in cases where the predicted variable takes its values in a restricted domain: the set of integer values, the domain of positive real numbers etc. The binomial regression is one case, and suits probabilities or a dependent categorical variable taking two values (Johnson, 2008; Morrison and Kondaurova, 2009). Considering the case of response times, Lo and Andrews (2015) explain how generalized models can come to the rescue of scholars facing two inappropriate choices: analyzing a raw dependent variable when this leads to violation of the assumptions of the linear model, or transforming this raw variable to meet these assumptions (as discussed in section “From Linear Regression Models to Mixed-Effects Linear Regression Models”). The appropriate generalized linear model offers a distribution of error terms leading to the satisfaction of assumptions without transformation. In addition to the conditional distribution of the dependent variable, a link function can also be specified; fixed factors can then linearly predict the result of the application of this function to the observed response, rather than the observed response itself. Among the more common link functions are the logarithm, square-root and inverse functions. Choosing link functions other than the identity function, however, leads once again to the evaluation of predictors with respect to a transformed dependent variable. When including random effects, GLM are usually called generalized linear mixed models, or GLMM.

The commonly available distributions in statistical packages dealing with GLM belong to the exponential family of distributions, such as the normal, Bernoulli, exponential, inverse-Gaussian, chi-squared, Poisson, or binomial (in this latter case, only when the number of trials is known) distributions.

*Phonemic inventory size* falls into the domain of count data, and it makes sense therefore to consider distributions over positive integers rather than over real numbers. The Poisson distribution is the better known option in such cases. In cases where the counts are small, i.e., close to 0, considering a distribution over real numbers would be dangerous, since predictions of the related model could be non-sensical negative values. A distribution over positive real numbers seems more appropriate, but exponential distributions like inverse-Gaussian or Gamma are not suited to count data close or equal to 0, unless in very specific cases. When count values are far from 0, however, continuous distributions may be considered, as it is the case for *Phonemic inventory size* – the smallest value is 11, the largest value 156, and the median 33. They may then give better results than discrete distributions. Given these considerations, we thus fitted to our data a Poisson regression, an inverse-Gaussian regression, and a Gamma regression, each time with an identity link function. This choice was motivated by the positive skewness of the distribution of inventory sizes. We used the *glmer()* function of the lme4 package (Bates et al., 2015), in which a few distributions of the exponential family can be specified, including the three previous ones. *glmer()* takes the same inputs as *lmer()* plus the chosen distribution.

The inverse-Gaussian distribution turned out to give the lowest deviance, which was much lower than that of the Poisson regression (10,693 vs. 11,653). The corresponding results (with restricted maximum likelihood – REML) are reported in **Table 2**. They depart from those of the previous LMM in that all the estimates for the fixed effects are closer to 0. The effect of *Distance from Africa* is still significant, but with a higher *p*-value, while *Number of speakers* and *Local linguistic density* are far from being significant. In addition to estimates for fixed predictors being closer to 0, all standard errors are smaller. This observation is a good point for the model.

**Table 2:** Output of an inverse-Gaussian GLMM applied to the data.

Predictors	Dependent variable			
	
	Number of phonemes			
	
	Estimate	Standard error	*z*-value	*p*-value
**Fixed parts**				
(Intercept)	35.65	2.33	15.26	**<0.001**
Distance from Africa	-3.52	1.36	-2.59	**0.010**
Number of speakers	-0.04	0.83	-0.05	0.957
Local linguistic density	0.15	0.62	0.18	0.855

**Random parts**				
τ_00,Family_	38.47			
*N*_Family_	139			
ICC_Family_	1.00			
Observations	1,529			
AIC	10,705			
Deviance	10,693			


**P-values lower than 0.05 are in bold*.*

**FIGURE 3:**
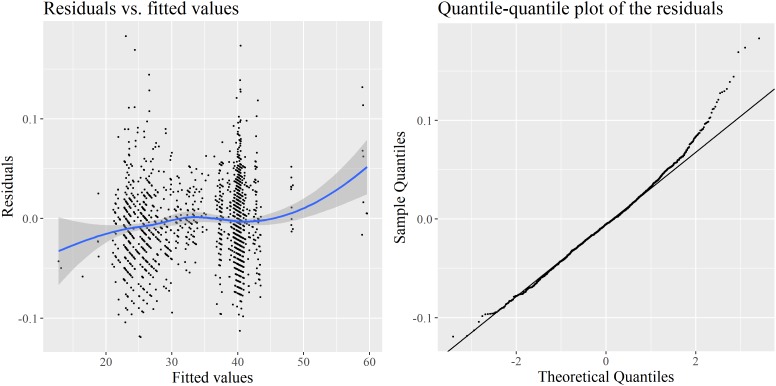
Diagnostics for the inverse-Gaussian GLMM: deviance residuals vs. fitted values **(left)** and quantile–quantile plot of these residuals **(right)**.

Again, a number of assumptions must be met for the output of the model to be acceptable. **Figure 3** contains two diagnostic plots for the inverse-Gaussian regression. Heteroscedasticity is more moderate than in the first LMM, but it appears that once again, the distribution of residuals departs from normality, although the problem is much less important than previously, as indicated by the range of sample values. The Gamma regression and the Poisson regression are not better in this respect. In the second case in particular, this is actually not surprising when one knows that the variance of the Poisson distribution is equal to its mean. The marginal distribution of *Phonemic inventory size* has a mean of 34.8, and a variance of 164.8: this is a clear case of strong overdispersion, which makes the Poisson distribution a very unlikely candidate for the regression.

### Generalized Additive Models (GAM)

Generalized additive models (GAM) are a family of models which were designed in the 1980s and are widely used today in a range of scientific fields (Hastie and Tibshirani, 1986). They are slowly making their way to linguistics, and a few authors recommend their use, for example in speech analysis (Sóskuthy, 2017).

Generalized additive models are at the intersection between additive models and generalized linear models. They are relevant when the relationship between a continuous predictor and the dependent variable is not adequately described by a linear regression (Wood, 2011; Winter and Wieling, 2016). Adopting a linear regression for a non-linear relationship is dangerous, since it creates autocorrelation patterns in the residuals, and therefore possibly unreliable estimates and confidence intervals for the model parameters (Sóskuthy, 2017). In some cases, non-linear relationships between a predictor and the dependent variable can be expressed by a simple polynomial of this predictor, and LMM or GLMM are then enough, but this is not always the case. GAM address this difficulty by allowing the presence of smoothing functions, or smoothers, in the linear predictor component of the regression model, along with “unsmoothed” covariates. The general equation of a GAM can thus be written:

$$g(E\left(Y\right))=I+s_{1}(x_{1})+...+s_{n}(x_{n})+\epsilon$$

where *x*_1_…*x_n_* are the predictors, *s*_1_(*x*_1_), …, *sn*(*xn*) the smooth terms relating to these predictors, *I* the intercept, ε the remaining error term, *Y* the dependent variable, *E*(*Y*) the expected value and *g* the link function.

The smooth terms can be either parametric (and this includes the linear and polynomial cases), semi-parametric or non-parametric, univariate or multivariate (in the latter case, to deal with interaction effects); they are overall very unconstrained and therefore very flexible. While this requires noticeably more observations, it can account for predictors and their influence more accurately. However, especially in the case of intricate non-linearities, interpreting the underlying causes can become much harder.

Among the more common parametric smoothers, one finds polynomials, fractional polynomials, piecewise polynomials, or B-splines. Non-parametric smoothers include local regression smoothers, such as the loess regression, which rely on a sliding window to extract local estimators, much in the way speech signals are analyzed to produce spectrograms. They also include penalized smoothers: for a single variable, cubic splines, P-splines, penalized B-splines, penalized categorical variables, Gaussian Markov random fields etc.; for several variables, thin plate regression splines, tensor product splines, varying coefficients etc. (Stasinopoulos et al., 2017, p. 257). While the differences between all these smoothers are beyond the scope of this article, it matters to say that the so-called penalization aims at finding the best value for the smoothing parameter, which controls the amount of smoothing, i.e., the degree of fitting of the smooth term to the raw predictor(s), unless this degree is specified externally by the user. The effective degrees of freedom (*edf*) can be referred to describe the amount of smoothing. The goal is here to avoid both underfitting and overfitting – the bias/variance tradeoff, so that the model can generalize well to data other than the sample used to build it.

Random effects can be included in GAM, in particular under the form of a specific penalized smoother (Stasinopoulos et al., 2017, p. 346). Random slopes can also be considered. One then speaks of generalized additive mixed models (GAMM), or “mixed GAM.” Significantly in a GAM(M), the smooth function of a predictor is estimated while taking into account all other predictors, whether smoothed or not.

In R, common packages for GAM(M) are gam, mgcv, or gamm4 (Wood, 2011), with differences in the underlying MLE algorithms. In mgcv, the function *gamm()* calls to the *lme()* function of the package nlme to estimate random effects, while *gamm4()* calls to *lmer()* or *glmer()*, all these secondary functions being related to LMM or GLMM. As said earlier, random effects can also be specified directly with a penalized smoothing function. It can be noted that the mgcv package enables the use of other distributions than those already mentioned, such as the Tweedie distribution, the zero-inflated Poisson distribution etc. (Wood et al., 2016).

Since the algorithms for MLE differ in GAM(M) and GLM(M), it makes sense to first check the output of an inverse-Gaussian GAM without smoothing functions. We used the *gam()* function of the mgcv package, with a random effect smoother for *Family*. **Table 3** gives the various elements of the model; the random effect clearly appears as a (very significant) smooth term. One can detect variations in the estimates, standard errors and *p*-values; in particular, the estimate for *Distance from Africa* is significantly larger than in the GLMM model. This illustrates the sensitivity of the results to the algorithm, and therefore reminds us to be cautious when concluding on the basis of only “slightly significant” *p*-values. As for GLMM, a Poisson GAM and a Gamma GAM both had higher deviance than the inverse-Gaussian GAM.

**Table 3:** Output of an inverse-Gaussian GAMM without smooth terms.

Predictors	Dependent variable			
	
	Number of phonemes			
	
	Estimate	Standard error	*z*-value	*p*-value
**Parametric coefficients**				
(Intercept)	36.25	2.21	16.43	**<0.001**
Distance from Africa	-5.20	1.29	-4.02	**<0.001**
Number of speakers	0.14	0.89	0.15	0.876
Local linguistic density	-0.08	0.83	-0.10	0.922

**Smooth term**	***edf***	***Ref.df***	***F***	***p*-value**

*s*(Family)	105	138	6.23	**<0.001**

Adjusted *R*^2^	0.416			
AIC	10,679			
Deviance explained	56.5%			


**P-values lower than 0.05 are in bold*.*

Looking back at the various relationships presented in **Figure 1**, several relationships between the predictors and *Phonemic inventory size* suggest that smooth terms may be relevant. The question, however, is whether the non-linear relationship observed on the surface between an isolated predictor and the dependent variable is intrinsic, or whether it is actually linear under the surface, but appears as non-linear due to the interlaced influence of other predictors. Considering several predictors and smooth terms in a single model allows one to disentangle the various influences at play. As a next step, we thus considered an inverse-Gaussian GAM with smoothers. Finding the most appropriate smoother(s) requires comparing different options and models with measures such as AIC or BIC, and it is generally advisable to estimate the smoothing parameter automatically, i.e., try a penalized version of the smoother. For the sake of simplicity here, we only compared two smoothers that we applied homogeneously to our three continuous fixed effects: cubic splines and P-splines. Regarding the former, the penalty was modified so as to shrink toward zero when the smoothing parameter goes to infinity. Concretely, this meant that an absence of relationship was correctly identified, i.e., with 0 effective degrees of freedom, rather than modeled with one degree of freedom as in standard cubic splines. We actually compared three approaches: penalized cubic splines, penalized P-splines, and cubic splines with a fixed smoothing parameter corresponding to two effective degrees of freedom, i.e., the minimum possible value, corresponding to polynomials of degree 2 (*k* = 3 in the specification of the model). Cubic splines and P-splines are common penalized smoothers, hence our choice; for more information on the differences between them, see (Stasinopoulos et al., 2017, p. 279).

**FIGURE 4:**
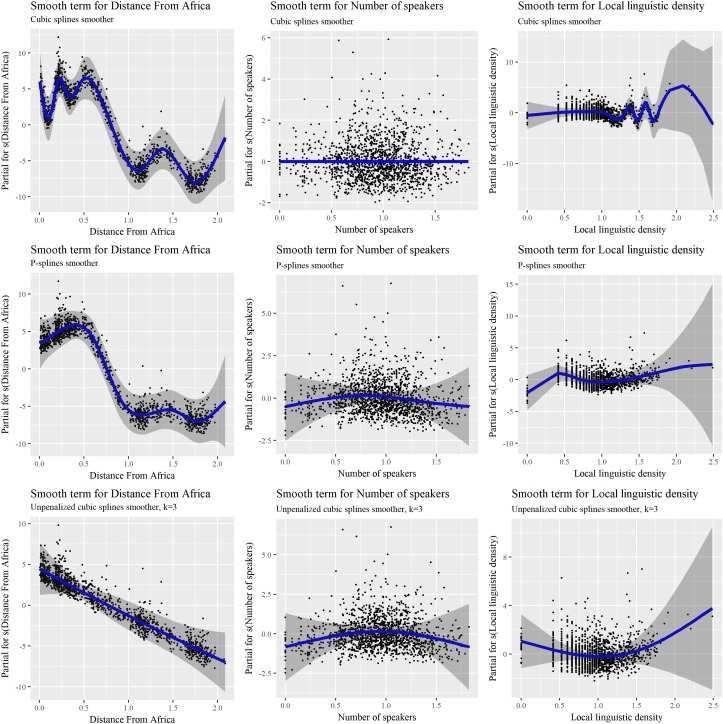
Smooth terms for *Distance from Africa*, *Number of Speakers*, and *Local linguistic density*, for three smoothing approaches in an inverse-Gaussian GAMM: cubic splines (top), P-splines (middle), and cubic splines with a fixed smoothing parameter equal to 3.

**Table 4** reports the outputs of the three models, and **Figure 4** the various smoothing terms for *Distance from Africa*, *Number of speakers*, and *Local linguistic density*. Regarding the numbers in **Table 4**, one should be careful with the standard errors and *p*-values reported for smooth terms. Indeed, these values are unreliable when the smoothing parameters have been penalized by the algorithm, because the uncertainty in the optimization of these parameters is not taken into account when assessing the null hypothesis. In consequence, *p*-values can be too low – again with potential type-I errors leading to falsely rejecting the null hypothesis. Likelihood ratio tests are more conservative than Wald chi-square tests, but results should still be examined with caution. A requirement in the presence of smoothing terms is to perform significance tests with un-penalized smooths, specifying the degree of smoothing as equal to the value obtained previously with penalization (Stasinopoulos et al., 2017, p. 125).

**Table 4:** Output of three inverse-Gaussian GAMM: with cubic splines for continuous predictors (top), with P-splines (middle), with cubic splines and a smoothing parameter fixed to 3 (bottom); in all models, a random effect smoother is applied to the predictor *Family*.

Predictors	Dependent variable			
	
	Number of phonemes			
	
Parametric coefficients	Estimate	Standard error	*z*-value	*p*-value
**Cubic splines, starting from *k* = 10**				
(Intercept)	33.58	1.15	29.12	**<0.001**

**Smooth terms**	***edf***	***Ref.df***	***F***	***p*-value**

*s*(Distance from Africa)	8.91	9.00	3017.56	**<0.001**
*s*(Number of speakers)	0.00	9.00	0.00	1.000
*s*(Local linguistic density)	8.72	9.00	9.98	0.325
*s*(Family)	101.69	138	3.87	**<0.001**

Adjusted *R*^2^	0.427			
AIC	10,649			
Deviance explained	57.9%			

**P-splines, starting from *k* = 10**				
(Intercept)	33.68	1.14	29.47	**<0.001**

**Smooth terms**	***edf***	***Ref.df***	***F***	***p*-value**

*s*(Distance from Africa)	5.82	6.52	6.67	**<0.001**
*s*(Number of speakers)	1.70	2.10	0.49	0.653
*s*(Local linguistic density)	4.47	5.20	1.52	0.207
*s*(Family)	100.70	138	3.66	**<0.001**

Adjusted *R*^2^	0.418			
AIC	10,662			
Deviance explained	57.2%			

**Cubic splines, *k* = 3**				
(Intercept)	32.37	1.13	28.55	**<0.001**

**Smooth terms**	***edf***	***Ref.df***	***F***	***p*-value**

*s*(Distance from Africa)	2	2	8.47	**<0.001**
*s*(Number of speakers)	2	2	0.42	0.655
*s*(Local linguistic density)	2	2	0.76	0.466
*s*(Family)	105.3	138	4.87	**<0.001**

Adjusted *R*^2^	0.416			
AIC	10,683			
Deviance explained	56.5%			


**P-values lower than 0.05 are in bold.**

The various graphs in **Figure 4** illustrate the subtleties of using GAM and choosing the right smoothers. As expected, unpenalized cubic splines smooth terms with a fixed number of two degrees of freedom result in relationships which display little “wiggliness”. In particular, they suggest a decreasing linear relationship between *Distance from Africa* and *Phonemic inventory size*, other predictors being accounted for. However, despite using less degrees of freedom (113.3 vs. 114.7 and 121.4 for penalized P-splines and cubic splines, respectively), the model has a higher AIC (10,682) than models with penalized P-splines and cubic splines (10,662 and 10,649, respectively). Contrary to what one could have expected, the degrees of freedom are actually only slightly lower than those of the two other models – with a difference of only 1.4 with the P-splines model. A closer look reveals that constraining the smoothness of continuous predictors is counterbalanced by more degrees of freedom used by the random effect *Family* (105.3 vs. 100.7 and 101.7 for penalized P-splines and cubic splines). Additionally, comparing the three models shows that 2 degrees of freedom is too much for *Number of* speakers: The penalized cubic splines model indicates an absence of relationship for this predictor (0 degrees of freedom), while the P-splines model returns 1.7 degrees of freedom. Altogether, these observations suggest that constraining the smooth terms to low degrees of freedom is not a very reasonable choice, and that the related model should rather be left aside. There is more generally no strong argument for choosing a priori 2 rather than 3 or 4 degrees, and penalizing the smooth term is a more neutral approach than starting by constraining the model with imprecise assumptions at the quantitative level.

Comparing now the two models with penalization, one sees that cubic splines lead to high degrees of non-linearity for *Distance from Africa* and *Local linguistic density*, which is reflected by the larger values of the effective degrees of freedom of these two smooth terms (8.90 and 8.72, respectively, to be compared to 5.82 and 4.47 for P-splines), while discarding an influence of *Number of speakers* (owing to the modified penalty introduced above). It looks as if canceling the influence of this predictor resulted in increased non-linearity in the two other continuous predictors. Different smooth functions thus result in different optimizations, something which is likely possible because of the complex correlations between *Distance from Africa*, *Number of speakers* and *Local linguistic density* (see **Figure 1**). Overall, the cubic splines model has the lowest AIC and should therefore be preferred in theory, although it does not provide any simple explanation for the shape of the non-linear relationship between for example *Distance from Africa* and *Phonemic inventory size*. While one may argue that the latter globally decreases with the former, things appear to be more complex than a linear relationship, and this while other predictors have been accounted for. P-splines lead to simpler smooth terms, but interpretation is still difficult. These results are interesting with respect to previous studies in the literature, which have always considered linear predictors rather than smooth terms. Some of the observed effects, as well as some of the contradictory results in different studies, may stem from an inappropriate modeling of non-linear relationships.

Does adding smooth terms to the regression model solve the issue of the non-normality of the residuals? In all previous GAMM models, residuals remain problematic, in a way very similar to those observed in **Figure 3** for the inverse-Gaussian GLMM. Previous observations with cubic splines and P-splines should therefore be treated with caution, and this calls for yet another modeling tool.

## Generalized Additive Models for Location, Scale, and Shape (GAMLSS)

### Overview

Generalized additive models for location, scale and shapes are an extension of GAM(M) which allows one to consider a wide range of options for the conditional distribution of the dependent variable, while GLM(M) and GAM(M) are restricted to the exponential family of distributions (Rigby and Stasinopoulos, 2005). Besides their range of values – all real numbers, positive real numbers, real numbers between 0 and 1 etc. –, distributions can be contrasted on the basis of their number of parameters: the Poisson distribution is defined with a single parameter, the Gaussian, Gamma, inverse-Gaussian distributions by two parameters etc. Some distributions, such as the generalized Gamma distribution – of which the Gamma and inverse-Gaussian distributions are two specific instances – or the exponential Gaussian distribution, rely on three parameters, while yet other distributions are defined by four parameters, such as the Johnson SU distribution. The terms location, scale, and shape refer to these various parameters, and are connected, but not necessarily equal, to the four moments of a distribution, namely the mean, the variance, the skewness, and the kurtosis. In the Poisson distribution, the single parameter is a location parameter, equal to the mean, and the scale and shape of the distribution are fixed – this corresponds to the fact that in a Poisson distribution, the variance is equal to the mean, the skewness to the square root of the mean, and the excess kurtosis (the kurtosis minus 3) to the inverse of the mean. In the Gaussian distribution, the mean and variance can be defined independently from each other and are the location and scale parameters, while the skewness and kurtosis, i.e., the shape, are both fixed, equal to the values 0 and 3, respectively. GAMLSS offer a large variety of distributions with 1, 2, 3, or 4 parameters, classically noted μ, σ, ν, and τ. While only μ is modeled in (G)LM(M) and GAM(M), in GAMLSS all four parameters can be modeled, either with linear parametric, non-linear parametric or non-parametric (smooth) functions of the predictors (Rigby et al., 2007). Normal random effects, but also non-parametric random effects can be considered. Mixtures of distributions can also be used. At the heart of the GAMLSS, algorithms have been designed to fill two tasks: maximize a penalized log-likelihood function addressing the estimates of fixed and random parameters, and evaluate the various smoothing parameters appropriately (Rigby et al., 2007; Stasinopoulos et al., 2017). These two operations cannot be disconnected, and various options are available to perform them in an imbricated way.

An example of the use of GAMLSS is given by Zha et al. (2016) in their analysis of motor vehicle crash data. The predicted variable consists in count data of crashes in highway segments in the United States over the course of several years. As previously stated, the Poisson regression is what usually comes first to mind when count data needs to be assessed. However, as seen for phonemic inventory size, the overdispersion is very high for the number of crashes. The negative binomial distribution better accounts for overdispersion, but by using GAMLSS, Zha et al. (2016) show that a Poisson-Inverse Gaussian provides a better fit and similar predictive performance. They thus suggest that it should be used in subsequent studies to obtain better estimates of the role of predictors. Another example is response times in psycholinguistic experiments. While Lo and Andrews (2015) report that inverse Gaussian and Gamma distributions are equivalent good fits for response times due to theoretical reasons, analysis of experimental data reveals that the distribution of residuals is not always satisfactory, especially because of the long tail of the distribution corresponding to long response times. Relying on distributions better accounting for the skewness of the target distribution, such as the generalized Gamma distribution, leads to more satisfying results in terms of normality of the residuals. Finally, Rigby et al. (2008) discuss various approaches to modeling overdispersed count data, among others 3-parameter Sichel and Delaporte distributions, as well as a 4-parameter distribution, the Poisson-shifted generalized inverse Gaussian distribution.

As for the overall philosophy of GAMLSS, it is interesting to quote Stasinopoulos et al. (2017, p. 26–27): “*GAMLSS provides greater flexibility in regression modeling, but with this flexibility comes more responsibility for the statistician. This is not a bad thing. The philosophy of GAMLSS is to allow the practitioner to have a wide choice of regression models.*”

In R, GAMLSS are available through several packages. The main package is named gamlss, but associated packages such as gamlss.add, gamlss.cens, gamlss.mx, gamlss.spatial etc. allow extending the main functionalities: generation of censored or truncated versions of the main distributions, additional smooth functions such as neural networks or decision trees, use of mixture distributions etc.

Models built with the aforementioned *lmer()*, *glmer()* or *gam()* functions can all be reproduced within the GAMLSS framework. Given the differences in the algorithms, outputs may, however, slightly differ from one model to the next.

### Investigating the Marginal Distribution of Phonemic Inventory Size

A first step in contemplating the use of GAMLSS to study phonemic inventory size is to pay a closer look at the distribution of the latter. The distribution of the dependent variable independently from any predictor is called the marginal distribution.

The *histDist()* and *fitDist()* functions of the gamlss package come in handy to investigate what theoretical distribution comes closest to the empirical one. The first one takes as its main inputs a vector of values and the name of a distribution, and returns how well the values fit the distribution, as expressed by the global deviance, the AIC and BIC of the fit. The second allows one to find the best fit among a list of distributions, and also returns the AIC of the different fitting attempts.

We used these two functions to compare different distributions. On the one hand, we considered distributions adapted to count data available in the gamlss.dist package (loaded by default with the gamlss package). There are over 25 available distributions, among them:

- The 1-parameter Poisson distribution (PO);
- The 2-parameter negative binomial distribution; the types I and II parametrizations (NBI and NBII) available in gamlss led to the same result, and we took the type I;
- The 2-parameter Poisson-Inverse Gaussian distribution (PIG);
- The 3-parameter Delaporte distribution (DEL);
- The 3-parameter Sichel distribution; we considered the second parametrization (SICHEL) offered in gamlss in order for the mean of the distribution to be equal to μ.

We also checked all the distributions adapted to positive real numbers. However, some distributions are based on parameters that are difficult to relate to the four moments mean, variance, skewness, and kurtosis. A location parameter, μ, equal to the mean of the distribution offers easier interpretations, and can be related to LMM, GLMM, and GAMM which all model the mean, and only the mean, of the distribution. This is the case for all previously reported discrete distributions (although with a specific parametrization for the Sichel distribution), but not for all continuous distributions – some of them, however, model the median, which is easy to interpret. Given this constraint of interpretability, we especially paid attention to:

- The 2-parameter inverse-Gaussian distribution (IG), following previous results with GLMM and GAM;
- The 3-parameter Generalized inverse-Gaussian (GIG), a generalization of IG with the mean as location parameter;
- The 3-parameter Box-Cox Cole and Green distribution (BCCG), with the median as location parameter;
- The 4-parameter Box-Cox *t* distribution (BCT), with the median as location parameter;
- The 4-parameter Box-Cox power exponential (BCPE), with the median as location parameter.

**FIGURE 5:**
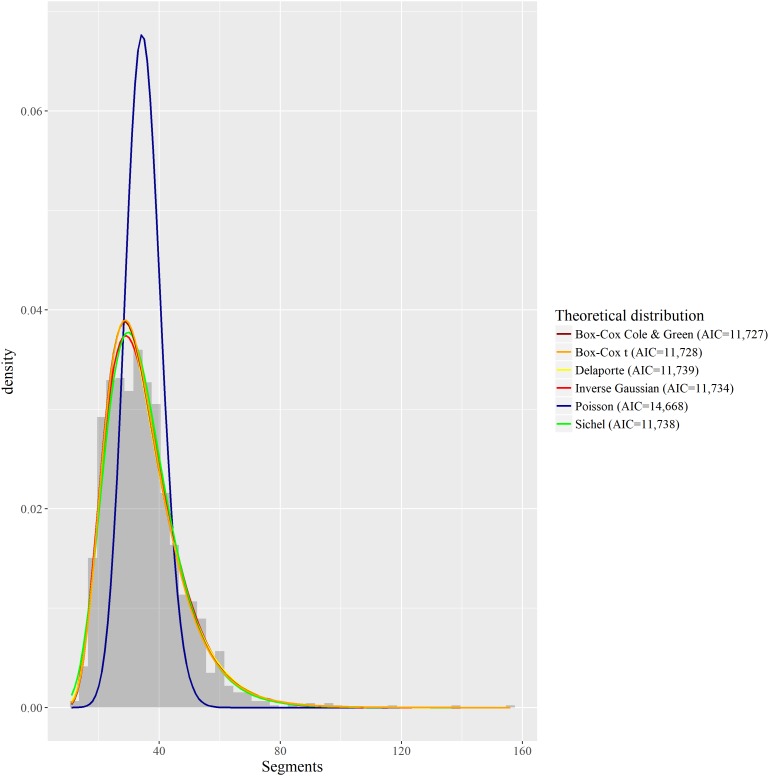
Fitting of several discrete and continuous theoretical distributions to the empirical distribution of *Phonemic inventory size*.

The intuition behind testing these various distributions was that those with more parameters would better be able to account for the thick right tail of the distribution, i.e., the positive skewness of this distribution. **Figure 5** summarizes the fits of the two most adequate discrete distributions, of the two most adequate continuous distributions, and of the Poisson and inverse-Gaussian distributions that were tested in previous models.

Among discrete distributions, the Sichel distribution has the lowest AIC (11,738), but is followed very closely by the Delaporte distribution (AIC = 11,739). The Poisson distribution has a much poorer fit (AIC = 14,668), which is in line with our previous results with GLMM and GAMM. Among continuous positive distributions, the BCCG distribution has the best fit in terms of AIC (11,727), followed by the Generalized Gamma (AIC = 11,727) which location cannot be easily related to the mean or median, and the BCT distribution (AIC = 11,728). The inverse-Gaussian distribution appears further away in the ranking (AIC = 11,734), but its distance to the best distributions is in no way comparable with how the Poisson distribution differs from the Sichel or Delaporte distributions. As visible on **Figure 5**, except for the Poisson distribution, all displayed theoretical distributions seem rather close to the empirical distribution. One can also observe here that strictly referring to AIC values, the BCCG and BCT distributions provide better fits that the SICHEL and DEL distributions.

Do these results suggest that the BCCG should be the distribution to use in a GAMLSS with our various predictors? One must be cautious here, since the marginal distribution is not the same as the conditional distribution of the dependent variable, i.e., its distribution when factoring in the various predictors. The question is whether the overdispersion can be explained by one or several of these predictors, or whether it is to some extent independent of them. In the second case, overdispersion will still be manifest in the conditional distribution, and will require treatment with a distribution with the right number of parameters. In the first case, given its degrees of freedom, this distribution will likely still provide good fitting. To this extent, the results obtained with the marginal distribution can serve as a guide in the choice of the target conditional distribution.

### Fitting a GAMLSS to Predict Phonemic Inventory Size

In practice, many decisions have to be made regarding the modeling options offered by GAMLSS, from choosing the distribution to choosing the link function, the additive terms and the smoothing parameters. Stasinopoulos et al. (2017, p. 380–384) provide valuable guidelines to operate adequate choices, although no strict sequence of operations can be followed blindly.

In our case, in the previous section, we first investigated the marginal distribution of the dependent variable to narrow down possible choices of distributions. Given the results, one can reasonably focus on a few distributions, namely the Sichel, Delaporte, Box-Cox Cole and Green, and Box-Cox *t* distributions. We also included the inverse-Gaussian distribution for the sake of comparison with previous models. Second, regarding the link function, we thought that keeping an identity link was useful to relate estimates of the models to actual number of phonemes, without the difficulties related to transforming the dependent variable – or the relationship between it and the predictors – as mentioned earlier in this article. Various link functions can actually be compared with AIC. In distributions requiring positive values, link functions such as the logarithm also prevent convergence issues that are otherwise difficult to address. Third, which additive terms to consider was like in all previous models related to current debates in the literature, which in no way means that other predictors would not be relevant. Various methods of model selection are available, some of them mixing backward, forward, and stepwise procedures across the various parameters of the distribution (Stasinopoulos et al., 2017, p. 385–402). However, besides the fact that some scholars disagree with the concept of model selection overall, the presence of a random effect for *Family* is somehow problematic. Indeed, the way this random effect is estimated in the model – a local normal approximation to likelihood, also known as penalized quasi likelihood – is different from what occurs in common LMM or GLMM – a global estimation to likelihood. The consequence is that dropping a continuous predictor can lead to a change in the penalization of the random effect, such that a strong effect, which should be retained by the selection procedure, may be abandoned. Because of this, we chose not to rely on selection procedures, but rather compare a number of models of increasing complexity. Thus, for each distribution, we considered a model with our predictors only for location (μ), a model with predictors additionally introduced for scale (σ), then, when possible, models with predictors additionally considered for shape parameters (ν then τ). As for smoothing finally, we considered P-splines smooth functions – cubic splines proved difficult to work with –, with a modified penalty so as to shrink toward zero when the smoothing parameter went to infinity – the *pbz()* smooth function in gamlss (Stasinopoulos et al., 2017, p. 274–275). The advantage of these smooth terms was that the estimation could lead to linear terms, or even to constant terms when no influence of a predictor was detected, other predictors being accounted for. Some parameter selection was thus present.

**Table 5** reports the deviance, the degrees of freedom used for the various parameters, the total number of used degrees of freedom, as well as the AIC and BIC of the various models tested. (DEL, μ, σ, and ν) refers for example to a model with the Delaporte distribution, and μ, σ, and ν modeled with our predictors. There were issues of convergence with Sichel models that we could not address, which explains why they are not discussed in what follows. In terms of deviance, the (BCT, μ, σ, and ν) and (BCT, μ, σ, ν, and τ) models had the lowest deviance. These two models were actually identical, which is explained by the fact that all predictors introduced to model τ ended up being estimated with 0 degrees of freedom – in other words, τ was best modeled with an intercept only. In terms of AIC, i.e., taking into account the number of degrees of freedom used by the models, the (DEL, μ, σ, and ν), (BCT, μ and σ) and (BCT, μ, σ, and ν) models were the best, with only a slight difference between them. Finally, the BIC pointed to the three Delaporte models as the most parsimonious.

**Table 5:** Comparisons of various GAMLSS models with different distributions and different levels of modeling of parameters.

Model	Global Deviance	*df* for μ	df for σ	*df* for ν	*df* for τ	*df*	AIC	BIC
IG, μ	10,450	104.47	1.00	0	0	105.47	10,661	11,224
IG, μ and σ	10,246	117.55	52.38	0	0	169.93	10,586	11,492
DEL, μ	10,456	79.73	1.00	1	0	81.73	10,619	**11,055**
DEL, μ and σ	10,356	83.24	20.42	1	0	104.66	10,565	**11,123**
DEL, μ, σ, and ν	10,344	83.14	18.86	4.00	0	106.00	**10,556**	**11,121**
BCCG, μ	10,424	105.68	1.00	1	0	107.68	10,640	11,214
BCCG, μ and σ	10,222	121.08	48.71	1	0	170.79	10,563	11,474
BCCG, μ, σ, and ν	10,219	121.38	49.42	3.00	0	173.80	10,567	11,494
BCT, μ	10,403	109.62	1.00	1	1	112.62	10,628	11,228
BCT, μ and σ	10,199	123.14	53.59	1	1	178.73	**10,557**	11,510
BCT, μ, σ, and ν	10,184	124.54	55.24	6.22	1	187.00	**10,558**	11,555
BCT, μ, σ, ν, and τ	10,184	124.54	55.24	6.22	1	187.00	**10,558**	11,555


**In each model, a penalized P-spline smooth function is used for the three continuous predictors, and a penalized random effect smoother for the categorical variable. The three lowest AIC and BIC are in bold. IG, inverse-Gaussian; DEL, Delaporte; BCCG, Box-Cox Green and Cole; and BCT, Box-Cox t**

**FIGURE 6:**
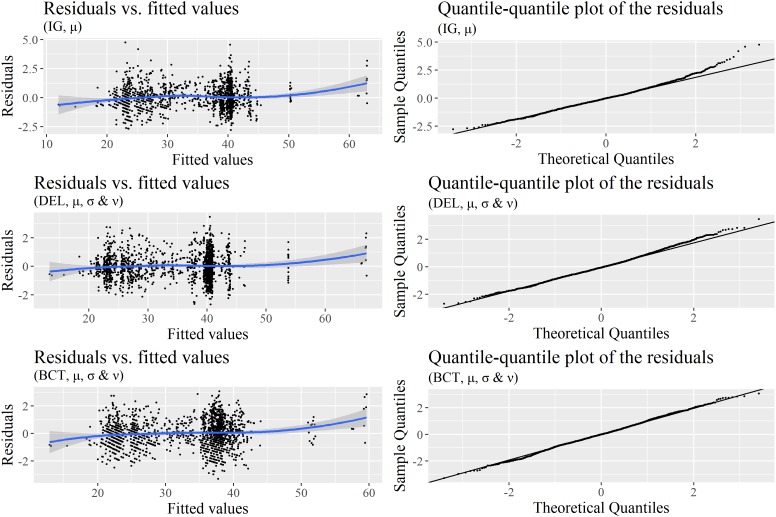
Diagnostics for the (IG, μ), (DEL, μ, σ, and ν), and (BCT, μ, σ, and ν) models reported in **Table 5**: Normalized quantile residuals vs. fitted values **(left)** and quantile-quantile plot of these residuals **(right)**.

**FIGURE 7:**
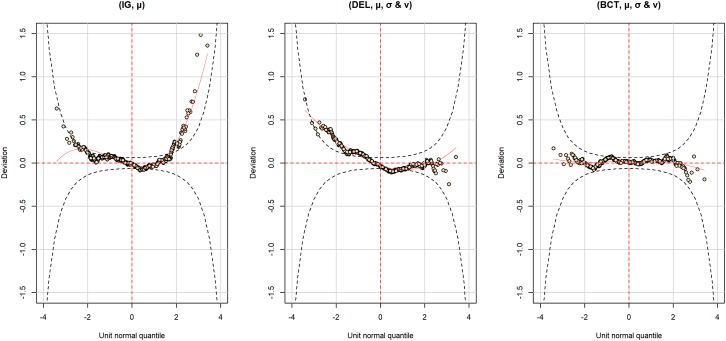
Detrended quantile–quantile plots – also known as worm plots – of the normalized quantile residuals for the (IG, μ), (DEL, μ, σ, and ν) and (BCT, μ, σ, and ν) models reported in **Table 5**.

Which of the previous models to choose, especially given the contradictions between the AIC and BIC? We first decided to prefer (BCT, μ, σ, and ν) over (BCT, μ and σ), since deviance was lower in the first model and since skewness could be better investigated with it. Checking an important assumption – the normality of the residuals – helped us to make a final choice between (BCT, μ, σ, and ν) and Delaporte models. **Figure 6** displays two diagnostic plots of the residuals – one to check homoscedasticity and the other to assess normality – for the (DEL, μ, σ, and ν) and (BCT, μ and σ) models, with (IG, μ) additionally as a reference. While, as previously seen, residuals strongly deviate from normality in (IG, μ), they are much better in (DEL, μ, σ, and ν) and (BCT, μ and σ). However, there is still some deviation in (DEL, μ, σ, and ν). **Figure 7**, which displays detrended quantile-quantile plots – also known as worm plots – provides a much clearer view of the problems of (IG, μ) and (DEL, μ, σ, and ν). In a worm plot, 95% of the dots must be within the 95% confidence interval defined by the two elliptic curves in the figure. This is not the case for the two models. By comparison, the residuals of the (BCT, μ and σ) model are very satisfying, which motivated our decision to adopt this model as the most relevant to further investigate our predictors.

Looking at the various effective degrees of freedom of the smooth terms, it appeared that many terms were actually equivalent to linear predictors, and the model could be simplified and described as follows:

- For μ, a smooth term is relevant for *Distance from Africa* and *Family*, and *Number of speakers* and *Local linguistic density* can be included without smoothing;
- For σ, a smooth terms is relevant for *Family*, and the three continuous predictors do not require smoothing;
- For ν, *Family* can be excluded, a smooth term is relevant for *Number of speakers*, but not for *Distance from Africa* and *Local linguistic density*.

**Table 6** reports the outputs of this model. Several predictors appear as statistically significant, however, Stasinopoulos et al. (2017, p. 18) warn that *p*-values should be inspected with caution when smooth terms are present. Indeed, the values given for a smooth term correspond to its linear part, and not to its total contribution. Additionally, reminiscent of what was said for GAM, the values for non-smoothed terms do not account for the uncertainty attached to the estimation of the smoothing terms. A partial solution to this problem is to consider likelihood-ratio tests to assess the significance of the predictors once the degrees of freedom of the smooth terms have been fixed to the values previously estimated with penalization (Stasinopoulos et al., 2017, p. 125). With such fixed smooth terms, dropping a predictor does not result in these smooth terms “reacting” to the drop by increasing their degrees of freedom. The *drop1()* function can be used to drop predictors one by one, whether in μ, σ, or ν, and obtain the *p*-value of the chi^2^ test involving the full model and the nested model without the dropped predictor (the difference in degrees of freedom is used for the test). **Table 7** reports the output of this function for our chosen model (described in **Table 6**).

**Table 6:** Output of a GAMLSS with (i) Box-Cox *t* distribution, (ii) μ, σ, and ν modeled with either linear predictors or penalized P-splines smooth functions of these predictors, and a penalized random effect smoother for the categorical variable *Family* when necessary, (iii) τ modeled as intercept only.

Predictors	Dependent variable			
	
	Number of phonemes			
	
Parametric coefficients	Estimate	Standard error	*t*-value	*p*-value
**μ (link function: identity)**				
(Intercept)	36.40	0.79	46.16	**<0.001**
*s*(Distance from Africa)	-6.05	0.30	-20.19	**<0.001**
Number of speakers	-0.19	0.52	-0.36	0.716
Local linguistic density	1.27	0.52	2.47	**0.014**
**σ (link function: log)**				
(Intercept)	-1.47	0.10	-15.16	**<0.001**
Distance from Africa	-0.26	0.04	-7.14	**<0.001**
Number of speakers	0.05	0.07	0.78	0.432
Local linguistic density	-0.14	0.07	-2.09	**0.037**
**ν (link function: identity)**				
(Intercept)	-1.00	0.51	-1.97	**0.048**
Distance from Africa	0.15	0.19	0.78	0.438
*s*(Number of speakers)	0.25	0.34	0.74	0.456
Local linguistic density	-0.02	0.30	-0.08	0.932
τ***(link function: log)***				
(Intercept)	11.97	3.39	3.53	**<0.001**

**Smooth terms**			**edf**	**σ*_B_***

**μ (link function: identity)**				
*s*(Distance from Africa)			8.83	1.08
*s*(Family)			113.71	6.96
**σ (link function: log)**				
*s*(Family)			51.23	0.31
**ν (link function: identity)**				
*s*(Number of speakers)			4.22	0.11

Global deviance	10,184			
AIC	10,558			
BIC	11,555			


*Regarding the parametric coefficients, the coefficient of a smoothing term and its standard error refer to its linear component. *P*-values lower than 0.05 are in bold.*

**Table 7:** Likelihood ratio tests (LRT) for the predictors of the (BCT, μ, σ, and ν) GAMLSS model described in **Table 6**.

	*df*	AIC	LRT	*p*-value
**μ**				
Starting model		10,558		
*s*(Distance from Africa)	7.82	10,571	29.28	**<0.001**
Number of speakers	1	10,556	0.22	0.640
Local linguistic density	1	10,560	4.11	**0.043**
*s*(Family)	113.71	10,874	544.30	**<0.001**
**σ**				
Starting model		10,558		
Distance from Africa	1	10,561	5.81	**0.016**
Number of speakers	1	10,556	0.12	0.726
Local linguistic density	1	10,561	5.39	**0.020**
*s*(Family)	51.23	10,600	145.24	**<0.001**
**ν**				
Starting model		10,558		
Distance from Africa	1	10,557	1.39	0.239
*s*(Number of speakers)	3.22	10,558	7.29	**0.074**
Local linguistic density	1	10,556	0.09	0.765


**P-values lower than 0.05 are in bold*.*

Regarding the median of the distribution, the smooth term for *Distance from Africa* is highly significant, while *Local linguistic density* is barely significant and *Number of speakers* is not. With τ constant, σ is approximately proportional to the coefficient of variation (the variance divided by the mean), and is significantly influenced by all predictors but *Number of speakers*. Finally, no predictor reaches the 0.05 significance threshold for ν. One can observe that for P-splines smooth terms, the difference in degrees of freedom between the full model and the model without the smooth term is equal to the fixed number of degrees of this smooth term minus 1. This is because the fixed number of degrees includes one degree for the intercept; when the smooth term is dropped, an intercept remains, hence the “minus 1.” One can also ponder here over the benefits of GAMLSS models which, in addition to predictions for the mean or median of the distribution, can also provide information regarding other moments of the distribution. In our case, a conclusion is that the coefficient of variation of the distribution significantly decreases as *Distance from Africa* increases, which means that inventories are more homogeneous in terms of size the further away from Africa, other factors being accounted for.

**FIGURE 8:**
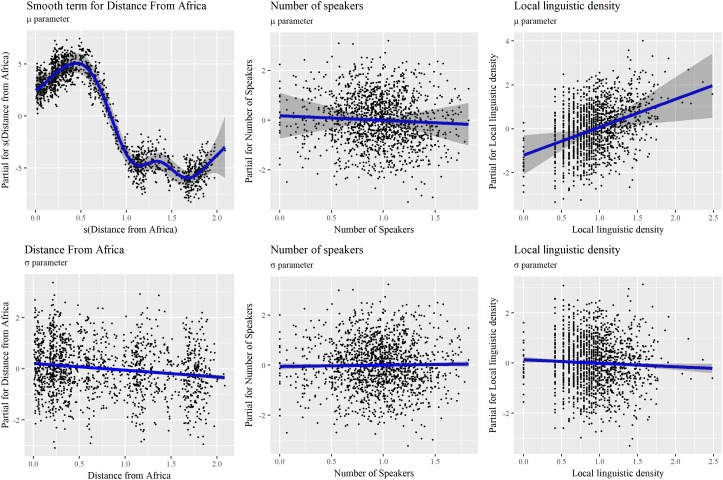
Additive terms for *Distance from Africa*, *Number of Speakers*, and *Local linguistic density* for the μ and σ components of the (BCT, μ, σ, and ν) model reported in **Tables 5**, **6**. A P-splines smooth function is applied to *Distance from Africa* for μ.

In order to better understand what is suggested by the model, it is necessary to look at the partial terms reproduced in **Figure 8**. The median of *Phonemic inventory size* is non-linearly related to *Distance from Africa*, and the two local maxima of the non-linear relation are not easy to interpret. As for GAMM, a linear decrease is not confirmed by the observed pattern. A sharp decrease can, however, be observed at some distance away from Africa. Relations for *Number of speakers* and *Local linguistic density* are linear. While the first one was assessed as not significant, the second one barely is, with an increase of the median *Phonemic inventory size* as the local linguistic density increases. This result was absent in previous LMM, GLMM, and GAMM models. This could be due to less satisfying statistical approaches, but should also serve as a warning of the limited trust one should put in this result.

## Discussion

Three aspects can be put forward in discussing the previous results and observations.

The first aspect concerns the specific nature of our target dependent variable, i.e., phonemic inventory size. The very large inventories of some languages, and the overdispersion of the connected variable, can be in good part explained by how features are combined into phonemes. The notion of feature economy states that “*speech sounds tend to be organized by a principle of feature economy, according to which languages maximize the combinatory possibilities of a few phonological features to generate large numbers of speech sounds*” (Clements, 2003, p. 371). According to this principle, very large inventories are so because some features are used intensively and produce series of phonemes “in mirror,” e.g., the vocalic feature of nasalization is put to use so that all vowels without secondary features have their nasalized counterparts. Multiplicative processes are therefore at the origin of at least some the variance and overdispersion of phonemic inventory size.

From this observation, one could argue that applying a transformation to the dependent variable makes sense, even if it is not an easy question to answer which transformation is respectful of the specific multiplicative processes at play. However, this transformation may run counter to the nature of the mechanisms hypothesized with the inclusion of a predictor. For example, referring to the impact of the number of speakers, does one conceive this impact at the level of phonemes, or at the level of features? In the latter case, the transformation would perhaps be justified. In the former, some situations could appear as less convincing. Although this hypothesis is far-fetched and is only put forward to the sake of argumentation, one could argue that having a larger number of speakers does not increase the number of features at the basis of the phonemic inventory, but rather influences the way speakers combine these features, in such a way that the system tends to display greater feature economy. Along the same line of thought, with respect to linguistic contact and the putative effect of the local linguistic density, the meaningful question would be whether speakers mostly borrow phonemes or features from other languages. In any case, one of the messages of this article is that models do exist that allow one to model “difficult” variables without resorting to transformation.

To move further in this direction, future work will consist in extracting the features of each phonemic inventory used in the test case of this article. It will then become possible to study the distribution of feature inventory size, much in the way phonemic inventory size was scrutinized in the previous sections. There are no multiplicative processes at the level of features, and it will therefore be relevant to evaluate the overdispersion of the marginal and conditional distributions. If overdispersion is still present and high, a possible conclusion will be that the overdispersion of phonemic inventory size derives from multiplicative processes when combining features, but also from the properties of the systems of features themselves.

A second point is the issue of weak effects in regression modeling. As it appears from our various analyses, *Distance from Africa* appears as a very significant effect in all models. One can assume that very strong and significant effects will be observed even with imperfect models. However, what about weaker effects, with significance close to the 5% threshold? Another predictors of our models, *Local linguistic density*, has *p*-values (well) above 0.05 in less satisfying models, and a *p*-value barely below 0.05 in the supposedly most appropriate model. Drawing conclusion about weak effects is very dependent on the model, especially if one clings to the 5% significance threshold, and also on the use of one test of significance over another: Wald *t*-tests, likelihood ratio tests, parametric bootstrapping etc. (Luke, 2017). On the one hand, some scientists advocate for moving away from the “null ritual” and the 5% threshold (Gigerenzer et al., 2004; Baker, 2016; Greenland et al., 2016; Wasserstein and Lazar, 2016), in which case differences between *p*-values slightly below or above 0.05 do not matter much. On the other hand, a conclusion is that weak signals are at the mercy of the chosen model, and thus this model should be chosen and assessed with care. For example, in the case of phonemic inventories, in addition to the assumptions we tested for residuals, potential spatial autocorrelation should be accounted for in order to minimize related type I errors. We have not addressed this concern in the previous models, but some options are available, whether it’s moving to regression models including spatial correlation structures, or including specific predictors such as the ‘weighted areal normalized phonological diversity’ proposed by Jaeger et al. (2011). All in all, with respect to our test case, whether language contact significantly affects phonemic inventory size through borrowing remains to us an open question. What geo-linguistic measures best capture language borrowing is a connected question that requires further investigation.

Finally, we argue that linguistics and psycholinguistics could benefit from the use of GAMLSS when regression models are envisaged to explore a phenomenon. The adequacy of the Delaporte distribution to model phonemic inventory size in no way means that this distribution in particular is *the* solution to a large number of problems. Rather, we have tried to highlight the reasoning that led us to consider this distribution, and why other options – LMM, GLMM, GAMM, GAMLSS with other distributions – were not as much appropriate. In other contexts, similar investigations would lead to another distribution or narrow choice of distributions. One domain of application already mentioned in Section “Overview” is the study of response times in psycholinguistics. In addition to finding appropriate theoretical distributions for the very specific distribution of reaction times (Moscoso Del Prado Martín, 2009; Baayen and Milin, 2010), a potentially fruitful advantage of GAMLSS is their ability to not only model mean, but also variance and skewness. Relating the mean of response times as dependent variable to a number of factors such as number of phonological neighbors, frequency, number of letters etc. is very common, but doing the same for the variance or the skewness could help further unravel the way cognitive treatment unfolds and linguistic information is processed.

Besides psycholinguistics, work in preparation suggests that another variable which can benefit from GAMLSS is speech rate. Indeed, speech rate – the number of syllables uttered by second – presents interesting variations between speakers and languages (Pellegrino et al., 2011; Coupé et al., 2014), but distributions in speakers and languages also suggest meaningful patterns of skewing, where the amount and orientation of skewing is connected to the mean value of the speech rate.

More generally, we have little doubt that many other variables, either continuous, discrete or count data, can benefit from both the smooth functions and distributions of GAMLSS.

## Conclusion

Various statistical tools are available to linguists willing to explain how a given linguistic variable varies across its domain. We highlighted how GAMLSS models, which are still very rarely used in the language sciences, could be put to use to depict ‘complex’ variables such as phonemic inventory size. This seems especially relevant when non-linguistic causes of linguistic diversity such as climatic or sociodemographic factors are considered, since their study can often be conducted with regression models. The distributions offered by GAMLSS can be more appropriate from a methodological point of view, and both the possibility to include additive terms and the possibility to model the scale and shape of the distribution in addition to its location can be put to use to better understand the behavior of a system.

## Data Availability Statement

The raw data supporting the conclusions of this manuscript will be made available by the author, without undue reservation, to any qualified researcher.

## Author Contributions

The author designed the work, assembled the studied dataset from other sources of data, conducted the different analyses, and wrote the article.

## Conflict of Interest Statement

The author declares that the research was conducted in the absence of any commercial or financial relationships that could be construed as a potential conflict of interest.
