# Supplementary material for: Modeling Linguistic Variables With Regression Models: Addressing Non-Gaussian Distributions, Non-independent Observations, and Non-linear Predictors With Random Effects and Generalized Additive Models for Location, Scale, and Shape
Source: Front Psychol. 2018 Apr 16;9:513. doi: 10.3389/fpsyg.2018.00513 (PMC5911484; doi:10.3389/fpsyg.2018.00513)
Supplement: Supplementary file 1 [file Presentation_1.PDF]

```r
# °°°°°°°°°°°°°°°°°°°°°°
# Loading libraries ####
# °°°°°°°°°°°°°°°°°°°°°°

library(car)
library(MASS) # for stdres

# Library for dataframe manipulation
library(reshape)
library(plyr)

# Libraries for generalized linear models (GLM/GLMM)
require(lme4) # For lmer and lme
require(lmerTest) # For the version of lmer with p-values

# Libraries for general additive models (GAM/GAMM)
library(mgcv)

# Libraries for GAMLSS
library(gamlss)
library(gamlss.mx)

# Graphical libraries
library(ggplot2)
library(GGally)
library(extrafont)
library(visreg)
library(gridExtra)


# °°°°°°°°°°°°°°°°°°°°°°°°°°°°°°°°°°°°°°°°°
# Preliminary definitions of functions ####
# °°°°°°°°°°°°°°°°°°°°°°°°°°°°°°°°°°°°°°°°°

get.figure.expected.values.vs.residuals <- function(model, subtitle = "") {
  df <- data.frame(x=predict(model), y=residuals(model))
  p <- ggplot(data=df, aes(x=x, y=y)) + geom_point(size=0.5) + geom_smooth(method="loess")
  p <- p + labs(title = "Residuals vs. fitted values", x = "Fitted values", y = "Residuals")
  if (subtitle != "")
    p <- p + labs(subtitle = subtitle)
  p <- p + theme(text = element_text(size=15, family="serif"))
  
  p
}

get.figure.predictor.vs.residuals <- function(model, predictor, predictor.name, subtitle = ""
) {
  
  df <- data.frame(x = predictor, y = residuals(model))
  p <- ggplot(data = df, aes(x = x, y = y)) + geom_point(size=0.5) + geom_smooth(method=
  "loess")
  p <- p + labs(title = paste0("Residuals vs. ", predictor.name), x = predictor.name, y = 
  "Residuals")
  if (subtitle != "")
    p <- p + labs(subtitle = subtitle)
  p <- p + theme(text = element_text(size=15, family="serif"))
  
```

```
  p
}

get.figure.qq.plot <- function(residuals, subtitle = "") {
  df <- data.frame(residuals=residuals)
  y <- quantile(residuals, c(0.25, 0.75))
  x <- qnorm(c(0.25, 0.75))
  slope <- diff(y)/diff(x)
  int <- y[1L] - slope * x[1L]
  
  p <- ggplot(data = df, aes(sample=residuals)) + stat_qq(size=0.5) + geom_abline(slope = 
  slope, intercept = int)
  p <- p + labs(title = "Quantile-quantile plot of the residuals", x = "Theoretical 
  Quantiles", y = "Sample Quantiles")
  if (subtitle != "")
    p <- p + labs(subtitle = subtitle)
  p <- p + theme(text = element_text(size=15, family="serif"))

  p
}

get.figure.partial.component <- function(model, predictor, predictor.name, smoother) {
  p <- visreg(model, predictor, gg=TRUE)
  p <- p + labs(title = paste0("Smooth term for ", predictor.name),
                subtitle = smoother, x = predictor.name, y = paste0("Partial for s(", 
                predictor.name, ")"))   
  p <- p + theme(text = element_text(size=10, family="serif"))
  
  p
}

get.results.gamlss <- function(m){
  switch(length(m$parameters),
         c(deviance(m), m$mu.df, 0, 0, 0, m$df.fit, AIC(m), BIC(m)),
         c(deviance(m), m$mu.df, m$sigma.df, 0, 0, m$df.fit, AIC(m), BIC(m)),
         c(deviance(m), m$mu.df, m$sigma.df, m$nu.df, 0, m$df.fit, AIC(m), BIC(m)),
         c(deviance(m), m$mu.df, m$sigma.df, m$nu.df, m$tau.df, m$df.fit, AIC(m), BIC(m)))
}

mydnbinom <- function(x, mu, sigma, log = FALSE) {
  dnbinom(x=x, size=1/sigma, mu=mu, log=log)
}


get.figure.partial.component.gam <- function(model, myData, colname, index, xlab, smoother) {
  pred.orig<-predict(model, type="terms")
  partial.resids<-pred.orig+residuals(model, type="scaled.pearson")
 
  col <- myData[,colname]
  tmp <- data.frame(x=col, y=partial.resids[,index])
  p <- ggplot(data=tmp, aes(x=x, y=y)) + geom_point(size=0.5) 
  p <- p + labs(title = paste0("Smooth term for ", xlab), subtitle = smoother, x = xlab, y = 
  paste0("Partial for s(", xlab, ")")) 
  
  pred.orig<-predict(model, type="terms", se.fit=T)
  fit<-pred.orig$fit[,index]
  fit.up95<-fit-1.96*pred.orig$se.fit[,index]
```

```
  fit.low95<-fit+1.96*pred.orig$se.fit[,index]

  or <- order(col)
  fit <- fit[or]
  fit.up95 <- fit.up95[or]
  fit.low95 <- fit.low95[or]

  tmp2 <- data.frame(x=col[or], y=fit, low95=fit.low95, up95=fit.up95)
  p <- p + geom_line(data=tmp2, aes(x=x, y=y), color="blue", size=1.5)
  p <- p + geom_ribbon(data=tmp2,aes(ymin=low95,ymax=up95),alpha=0.3)
  p <- p + theme(text = element_text(size=10, family="serif"))

  p
}


get.figure.partial.component.gamlss <- function(model, myData, colname, index, title, xlab,
what="mu") {
  pred.orig<-predict(model, what=what, type="terms")
  partial.resids<-pred.orig+residuals(model, type="partial")

  col <- myData[,colname]
  tmp <- data.frame(x=col, y=partial.resids[,index])
  p <- ggplot(data=tmp, aes(x=x, y=y)) + geom_point(size=0.5)

  if (what == "mu")
    p <- p + labs(title = title,
                  subtitle = expression(mu*" parameter"), x = xlab, y = paste0("Partial for "
                  , xlab))

  if (what == "sigma")
    p <- p + labs(title = title,
                  subtitle = expression(sigma*" parameter"), x = xlab, y = paste0("Partial
                  for ", xlab))

  if (what == "nu")
    p <- p + labs(title = title,
                  subtitle = expression(nu*" parameter"), x = xlab, y = paste0("Partial for "
                  , xlab))

  if (what == "tau")
    p <- p + labs(title = title,
                  subtitle = expression(tau*" parameter"), x = xlab, y = paste0("Partial for
                  ", xlab))

  pred.orig<-predict(model, what=what, type="terms", se=T)
  fit<-pred.orig$fit[,index]
  fit.up95<-fit-1.96*pred.orig$se.fit[,index]
  fit.low95<-fit+1.96*pred.orig$se.fit[,index]

  or <- order(col)
  fit <- fit[or]
  fit.up95 <- fit.up95[or]
  fit.low95 <- fit.low95[or]

  tmp2 <- data.frame(x=col[or], y=fit, low95=fit.low95, up95=fit.up95)
  p <- p + geom_line(data=tmp2, aes(x=x, y=y), color="blue", size=1.5)
```

```
  p <- p + geom_ribbon(data=tmp2,aes(ymin=low95,ymax=up95),alpha=0.3)
  p <- p + theme(text = element_text(size=10, family="serif"))
  
  p
}


# °°°°°°°°°°°°°°°°°°°°°°°°°°°°
# Loading windows fonts ####
# °°°°°°°°°°°°°°°°°°°°°°°°°°°°

loadfonts(device = "win")
windowsFonts()


# °°°°°°°°°°°°°°°°°°
# Loading data ####
# °°°°°°°°°°°°°°°°°°

folder <- "D:/"

lg.df = read.table(paste0(folder, "Data.txt"), header = TRUE, sep = "\t", dec = ".", quote =
"")


# °°°°°°°°°°°°°°°°°°°°
# Curating data #####
# °°°°°°°°°°°°°°°°°°°°

# Transforming continuous predictors
lg.df$DistanceFromAfrica <- as.vector(scale(lg.df$DistanceFromAfrica, center=F))
lg.df$NbSpeakers <- as.vector(scale(lg.df$logNbSpeakers, center=F))
lg.df$LgDensity <- as.vector(scale(lg.df$LgDensity^(1/3), center=F))

summary(lg.df)

# Modifying the families of creole languages, so that these languages do not fall into the
same "Creole" family
nb.creoles <- nrow(lg.df[lg.df$Family == "Creole",])
new.creole.families <- paste0("Creole ", c(1:nb.creoles))
lg.df$Family <- as.character(lg.df$Family)
lg.df[lg.df$Family == "Creole",]$Family <- new.creole.families
lg.df$Family <- as.factor(lg.df$Family)
levels(lg.df$Family)

# Computing the number of languages in the different families
lg.df.cast = melt(lg.df, id=c("Family"), measure="Iso")
(family.sizes <- cast(lg.df.cast, Family ~ ., length))


# °°°°°°°°°°°°°°°°°°°°°°°°°°
# Info about segments ####
# °°°°°°°°°°°°°°°°°°°°°°°°°°

mean(lg.df$Segments)
median(lg.df$Segments)
var(lg.df$Segments)
```

```
min(lg.df$Segments)
max(lg.df$Segments)


# °°°°°°°°°°°°°°°°°°°°°°°°°°°°°°°°°°°°°°°°°°°°°°°°°°°°°°°°°°°°°°°°°°°°°°°°°°°°°°
# Displaying relationships among the predictors and the predicted variable ####
# °°°°°°°°°°°°°°°°°°°°°°°°°°°°°°°°°°°°°°°°°°°°°°°°°°°°°°°°°°°°°°°°°°°°°°°°°°°°°°

gg.fn <- function(data, mapping, ...){
  p <- ggplot(data = data, mapping = mapping) + geom_point(size=1) +
      geom_smooth(method=loess, fill="red", color="red", ...) + geom_smooth(method=lm, fill=
      "blue", color="blue", ...)
  p
}

my.gg.pairs <- ggpairs(lg.df[,c("NbSpeakers", "LgDensity", "DistanceFromAfrica", "Segments")],
            lower = list(continuous = gg.fn),
            upper = list(continuous = wrap("cor", size = 5, family="serif")),
            columnLabels = c("Nb of speakers", "Local linguistic density", "Distance from
            Africa", "Nb of phonemes")) +
            theme(text = element_text(size=16, family="serif"))
my.gg.pairs

ggsave("GGPAIRS.tiff", plot = my.gg.pairs, device = 'tiff', path = folder,
      scale = 1, width = 85*3.125, height = 85*3.125, units = "mm", dpi = 300, compression =
       "lzw")

# °°°°°°°°°°°°°°°°°°°°°°°°°°°°°°°°°°°°°°°°°°°°°°°°°°°°°°°°°°°°°°°°°°°°°°°°°°°°°°°°°
# checking the variance inflation factor (VIF) of our three linear predictors ####
# °°°°°°°°°°°°°°°°°°°°°°°°°°°°°°°°°°°°°°°°°°°°°°°°°°°°°°°°°°°°°°°°°°°°°°°°°°°°°°°°°

vif(lg.df[,c("NbSpeakers", "LgDensity", "DistanceFromAfrica")])

# °°°°°°°°°°°°°°°
# LMER model ####
# °°°°°°°°°°°°°°°

model.lmer <- lmer(Segments ~ DistanceFromAfrica + NbSpeakers + LgDensity + (1|Family), data
= lg.df)
summary(model.lmer)
AIC(model.lmer)

(fig1 <- get.figure.expected.values.vs.residuals(model.lmer))
(fig2 <- get.figure.qq.plot(residuals(model.lmer)))
(fig3 <- get.figure.qq.plot(ranef(model.lmer)$Family[,1]))

merged.figures <- arrangeGrob(fig1, fig2, nrow=1, ncol=2)
plot(merged.figures)

ggsave("LMER.tiff", plot = merged.figures, device = 'tiff', path = folder,
      scale = 1, width = 85*3.125, height = 85*3.125/2, units = "mm", dpi = 300, compression
       = "lzw")

# Displaying additional figures to assess the linearity of the predictions
get.figure.predictor.vs.residuals(model.lmer, lg.df$DistanceFromAfrica, "Distance from
Africa")
get.figure.predictor.vs.residuals(model.lmer, lg.df$NbSpeakers, "Nb of speakers")
```

```
get.figure.predictor.vs.residuals(model.lmer, lg.df$LgDensity, "Linguistic density")


# °°°°°°°°°°°°°°°°°°°°°°°°°°°°°°°°°
# LMER with log transformation ####
# °°°°°°°°°°°°°°°°°°°°°°°°°°°°°°°°°

model.lmer.log <- lmer(log(Segments) ~ DistanceFromAfrica + NbSpeakers + LgDensity + (1|
Family), data = lg.df)
summary(model.lmer.log)
AIC(model.lmer.log)

(fig1 <- get.figure.expected.values.vs.residuals(model.lmer.log))
(fig2 <- get.figure.qq.plot(residuals(model.lmer.log)))
(fig3 <- get.figure.qq.plot(ranef(model.lmer.log)$Family[,1]))

merged.figures <- arrangeGrob(fig1, fig2, nrow=1, ncol=2)
plot(merged.figures)

ggsave("LMER.LOG.tiff", plot = merged.figures, device = 'tiff', path = folder,
       scale = 1, width = 85*3.125, height = 85*3.125/2, units = "mm", dpi = 300, compression
       = "lzw")

# °°°°°°°°°°°°°°°°°°
# GLMER, Poisson ####
# °°°°°°°°°°°°°°°°°°

model.glmer.Poisson <- glmer(Segments ~ DistanceFromAfrica + NbSpeakers + LgDensity + (1|
Family), 
                             family=poisson(link="identity"), data = lg.df)
summary(model.glmer.Poisson)
AIC(model.glmer.Poisson)

(fig1 <- get.figure.expected.values.vs.residuals(model.glmer.Poisson))
(fig2 <- get.figure.qq.plot(residuals(model.glmer.Poisson)))
(fig3 <- get.figure.qq.plot(ranef(model.glmer.Poisson)$Family[,1]))

merged.figures <- arrangeGrob(fig1, fig2, nrow=1, ncol=2)
plot(merged.figures)

ggsave("GLMER.POISSON.tiff", plot = merged.figures, device = 'tiff', path = folder,
       scale = 1, width = 85*3.125, height = 85*3.125/2, units = "mm", dpi = 300, compression
       = "lzw")


# °°°°°°°°°°°°°°°°°°°°°°°°°°°°°°°°°°°
# GLMER, Gamma & inverse Gaussian ####
# °°°°°°°°°°°°°°°°°°°°°°°°°°°°°°°°°°°

model.glmer.GA <- glmer(Segments ~ DistanceFromAfrica + NbSpeakers + LgDensity + (1|Family), 
                        family=Gamma(link="identity"), data = lg.df)
summary(model.glmer.GA)
AIC(model.glmer.GA)

(fig1 <- get.figure.expected.values.vs.residuals(model.glmer.GA))
(fig2 <- get.figure.qq.plot(residuals(model.glmer.GA)))
(fig3 <- get.figure.qq.plot(ranef(model.glmer.GA)$Family[,1]))
```

```
model.glmer.IG <- glmer(Segments ~ DistanceFromAfrica + NbSpeakers + LgDensity + (1|Family), 
                         family=inverse.gaussian(link="identity"), data = lg.df)
summary(model.glmer.IG)
AIC(model.glmer.IG)

(fig1 <- get.figure.expected.values.vs.residuals(model.glmer.IG))
(fig2 <- get.figure.qq.plot(residuals(model.glmer.IG)))
(fig3 <- get.figure.qq.plot(ranef(model.glmer.IG)$Family[,1]))


merged.figures <- arrangeGrob(fig1, fig2, nrow=1, ncol=2)
plot(merged.figures)

ggsave("GLMER.IG.tiff", plot = merged.figures, device = 'tiff', path = folder, 
       scale = 1, width = 85*3.125, height = 85*3.125/2, units = "mm", dpi = 300, compression
       = "lzw")


# °°°°°°°°°°°°°°°°°°°°°°°°°°°°°°°°°°°°°°
# GAM, Inverse Gaussian, no smoother ####
# °°°°°°°°°°°°°°°°°°°°°°°°°°°°°°°°°°°°°°

model.gam.Poisson <- gam(Segments ~ DistanceFromAfrica + NbSpeakers + LgDensity 
                          + s(Family,bs="re"), family = poisson(link="identity"), data = 
                          lg.df)
summary(model.gam.Poisson)
AIC(model.gam.Poisson)


model.gam.IG <- gam(Segments ~ DistanceFromAfrica + NbSpeakers + LgDensity 
                     + s(Family,bs="re"), family = inverse.gaussian(link="identity"), data = 
                     lg.df)
summary(model.gam.IG)
AIC(model.gam.IG)

(fig.expected.res.gam.linear <- get.figure.expected.values.vs.residuals(model.gam.IG))
(fig.qq.residuals.gam.linear <- get.figure.qq.plot(residuals(model.gam.IG)))

(fig.pred1.gam.linear <- get.figure.partial.component.gam(model.gam.IG, lg.df, 
"DistanceFromAfrica", 1, "Distance From Africa", "No smoother"))
(fig.pred2.gam.linear <- get.figure.partial.component.gam(model.gam.IG, lg.df, "NbSpeakers", 
2, "Number of speakers", "No smoother"))
(fig.pred3.gam.linear <- get.figure.partial.component.gam(model.gam.IG, lg.df, "LgDensity", 3
, "Local linguistic density", "No smoother"))

# °°°°°°°°°°°°°°°°°°°°°°°°°°°°°°°°°°°°°°°°
# GAM, Inverse Gaussian, cubic splines ####
# °°°°°°°°°°°°°°°°°°°°°°°°°°°°°°°°°°°°°°°°

model.gam.IG.cs <- gam(Segments ~ s(DistanceFromAfrica, bs="cs") + s(NbSpeakers, bs="cs") + s
(LgDensity, bs="cs") 
                        + s(Family,bs="re"), family=inverse.gaussian(link="identity"), data = 
                        lg.df)

summary(model.gam.IG.cs)
```

```
AIC(model.gam.IG.cs)

(fig.expected.res.gam.cs <- get.figure.expected.values.vs.residuals(model.gam.IG.cs))
(fig.qq.residuals.gam.cs <- get.figure.qq.plot(residuals(model.gam.IG.cs)))

(fig.pred1.gam.cs <- get.figure.partial.component.gam(model.gam.IG.cs, lg.df, 
"DistanceFromAfrica", 1, "Distance From Africa", "Cubic splines smoother"))
(fig.pred2.gam.cs <- get.figure.partial.component.gam(model.gam.IG.cs, lg.df, "NbSpeakers", 2
, "Number of speakers", "Cubic splines smoother"))
(fig.pred3.gam.cs <- get.figure.partial.component.gam(model.gam.IG.cs, lg.df, "LgDensity", 3
, "Local linguistic density", "Cubic splines smoother"))


# °°°°°°°°°°°°°°°°°°°°°°°°°°°°°°°°°°°°°
# GAM, Inverse Gaussian, P-splines ####
# °°°°°°°°°°°°°°°°°°°°°°°°°°°°°°°°°°°°°

model.gam.IG.ps <- gam(Segments ~ s(DistanceFromAfrica, bs="ps") + s(NbSpeakers, bs="ps") + s
(LgDensity, bs="ps") 
                       + s(Family,bs="re"), family=inverse.gaussian(link="identity"), data = 
                       lg.df)

summary(model.gam.IG.ps)
AIC(model.gam.IG.ps)

(fig.expected.res.gam.ps <- get.figure.expected.values.vs.residuals(model.gam.IG.ps))
(fig.qq.residuals.gam.ps <- get.figure.qq.plot(residuals(model.gam.IG.ps)))

(fig.pred1.gam.ps <- get.figure.partial.component.gam(model.gam.IG.ps, lg.df, 
"DistanceFromAfrica", 1, "Distance From Africa", "P-splines smoother"))
(fig.pred2.gam.ps <- get.figure.partial.component.gam(model.gam.IG.ps, lg.df, "NbSpeakers", 2
, "Number of speakers", "P-splines smoother"))
(fig.pred3.gam.ps <- get.figure.partial.component.gam(model.gam.IG.ps, lg.df, "LgDensity", 3
, "Local linguistic density", "P-splines smoother"))

# °°°°°°°°°°°°°°°°°°°°°°°°°°°°°°°°°°°°°°°°°°°°°°°°°°°°°
# GAM, Inverse Gaussian, cubic splines, k=3 (edf=2) ####
# °°°°°°°°°°°°°°°°°°°°°°°°°°°°°°°°°°°°°°°°°°°°°°°°°°°°°

model.gam.IG.cs.fx <- gam(Segments ~ s(DistanceFromAfrica, bs="cs", k=3, fx=T) 
                          + s(NbSpeakers, bs="cs", k=3, fx=T) 
                          + s(LgDensity, bs="cs", k=3, fx=T) 
                          + s(Family,bs="re"), family=inverse.gaussian(link="identity"), data
                           = lg.df)

summary(model.gam.IG.cs.fx)
AIC(model.gam.IG.cs.fx)

(fig.expected.res.gam.cs.fx <- get.figure.expected.values.vs.residuals(model.gam.IG.cs.fx))
(fig.qq.residuals.gam.cs.fx <- get.figure.qq.plot(residuals(model.gam.IG.cs.fx)))

(fig.pred1.gam.cs.fx <- get.figure.partial.component.gam(model.gam.IG.cs.fx, lg.df, 
"DistanceFromAfrica", 1, "Distance From Africa", "Unpenalized cubic splines smoother, k=3"))
(fig.pred2.gam.cs.fx <- get.figure.partial.component.gam(model.gam.IG.cs.fx, lg.df, 
"NbSpeakers", 2, "Number of speakers", "Unpenalized cubic splines smoother, k=3"))
(fig.pred3.gam.cs.fx <- get.figure.partial.component.gam(model.gam.IG.cs.fx, lg.df, 
"LgDensity", 3, "Local linguistic density", "Unpenalized cubic splines smoother, k=3"))
```

```
# °°°°°°°°°°°°°°°°°°°°°°°°°°°
# GAM, Graphical outputs ####
# °°°°°°°°°°°°°°°°°°°°°°°°°°°

merged.figures <- arrangeGrob(fig.pred1.gam.cs, fig.pred2.gam.cs, fig.pred3.gam.cs,
                  fig.pred1.gam.ps, fig.pred2.gam.ps, fig.pred3.gam.ps,
                  fig.pred1.gam.cs.fx, fig.pred2.gam.cs.fx, fig.pred3.gam.cs.fx,
                  nrow=3, ncol=3)
plot(merged.figures)

ggsave("GAMs.tiff", plot = merged.figures, device = 'tiff', path = folder,
       scale = 1, width = 85*3.125, height = 85*3.125, units = "mm", dpi = 300, compression =
       "lzw")


# °°°°°°°°°°°°°°°°°°°°°°°°°°°°°°°°°°°°°°°°°°°°°°°°°°°°°°°°°°°°°°°°°°°°°°°°°°°°°°°
# GAMLSS, Approaching the marginal distribution of phonemic inventory size ####
# °°°°°°°°°°°°°°°°°°°°°°°°°°°°°°°°°°°°°°°°°°°°°°°°°°°°°°°°°°°°°°°°°°°°°°°°°°°°°°°

test.distributions.real <- fitDist(lg.df$Segments, k = 2, type = "realAll")
test.distributions.counts <- fitDist(lg.df$Segments, k = 2, type = "counts")

as.data.frame(test.distributions.real$fits)
as.data.frame(test.distributions.counts$fits)

nb_interpol <- max(lg.df$Segments) - min(lg.df$Segments) + 1

(mPO <- histDist(lg.df$Segments, "PO", density = TRUE, ylim = c(0,0.07), main = "Poisson
distribution"))
(mNBI <- histDist(lg.df$Segments, "NBI", density = TRUE, ylim = c(0,0.07), main = "Negative
binomial distribution"))
(mPIG <- histDist(lg.df$Segments, "PIG", density = TRUE, ylim = c(0,0.07), main = "Poisson
inverse Gaussian distribution"))
(mDEL <- histDist(lg.df$Segments, "DEL", density = TRUE, ylim = c(0,0.07), main = "Delaporte
distribution"))
(mSI <- histDist(lg.df$Segments, "SICHEL", density = TRUE, ylim = c(0,0.07), main = "Sichel
distribution"))
(mIG <- histDist(lg.df$Segments, "IG", density = TRUE, ylim = c(0,0.07), main = "Inverse
Gaussian distribution"))
(mGIG <- histDist(lg.df$Segments, "GIG", density = TRUE, ylim = c(0,0.07), main =
"Generalized inverse Gaussian distribution"))
(mBCCG <- histDist(lg.df$Segments, "BCCG", density = TRUE, ylim = c(0,0.07), main = "Box-Cox
Cole & Green distribution"))
(mBCT <- histDist(lg.df$Segments, "BCT", density = TRUE, ylim = c(0,0.07), main = "Box-Cox t
distribution"))

comparison.distributions <- ggplot(lg.df,aes(x=Segments)) +
  geom_histogram(aes(y=..density..), binwidth = 3, fill = "black", alpha = 0.2) +
  stat_function(fun = dpois, args = list(mPO$mu, FALSE), n=nb_interpol, size = 0.8, aes(
  colour = "Poisson (AIC=14,668)")) +
  stat_function(fun = dIG, args = list(mIG$mu, mIG$sigma, FALSE), n=nb_interpol, size = 0.8,
  aes(colour = "Inverse Gaussian (AIC=11,734)")) +
  stat_function(fun = dDEL, args = list(mDEL$mu, mDEL$sigma, mDEL$nu, FALSE), n=nb_interpol,
  size = 0.8, aes(colour = "Delaporte (AIC=11,739)")) +
  stat_function(fun = dSICHEL, args = list(mSI$mu, mSI$sigma, mSI$nu, FALSE), n=nb_interpol,
```

```
  size = 0.8, aes(colour = "Sichel (AIC=11,738)")) +
  stat_function(fun = dBCCG, args = list(mBCCG$mu, mBCCG$sigma, mBCCG$nu, FALSE), n=
  nb_interpol, size = 0.8, aes(colour = "Box-Cox Cole & Green (AIC=11,727)")) +
  stat_function(fun = dBCT, args = list(mBCT$mu, mBCT$sigma, mBCT$nu, mBCT$tau, FALSE), n=
  nb_interpol, size = 0.8, aes(colour = "Box-Cox t (AIC=11,728)")) +
  scale_colour_manual("Theoretical distribution", values = c("darkred", "orange", "yellow",
  "red", "darkblue", "green")) +
  theme(text = element_text(size=15, family="serif"))

comparison.distributions

ggsave("GAMLSS_MARGINAL_DISTRIBUTIONS.tiff", plot = comparison.distributions, device = 'tiff'
, path = folder,
       scale = 1, width = 85*3.125, height = 85*3.125, units = "mm", dpi = 300, compression =
        "lzw")


# °°°°°°°°°°°°°°°°°°°°°°°°°°°°°°°°°°°°°°°°°°°°°°°°°°°°°°°°°°°°°°°°°°°°°°
# GAMLSS, Testing various models with IG, SICHEL, DEL, BCCG and BCT ####
# °°°°°°°°°°°°°°°°°°°°°°°°°°°°°°°°°°°°°°°°°°°°°°°°°°°°°°°°°°°°°°°°°°°°°°

# IG
model.gamlss.IG.empty <- gamlss(Segments ~ 1, data = lg.df, family=IG(mu.link="identity"),
mu.start = 38,
                                 control = gamlss.control(n.cyc = 200), i.control=
                                 glim.control(bf.cyc = 200))
model.gamlss.IG.mu <- update(model.gamlss.IG.empty, ~ pbz(DistanceFromAfrica) + pbz(
NbSpeakers) + pbz(LgDensity) + random(Family))
model.gamlss.IG.sigma <- update(model.gamlss.IG.mu, ~ pbz(DistanceFromAfrica) + pbz(
NbSpeakers) + pbz(LgDensity) + random(Family), what="sigma")

# DEL
model.gamlss.DEL.empty <- gamlss(Segments ~ 1, data = lg.df, family=DEL(mu.link="identity"),
mu.start = 38,
                                 control = gamlss.control(n.cyc = 200), i.control=
                                 glim.control(bf.cyc = 200))
model.gamlss.DEL.mu <- update(model.gamlss.DEL.empty, ~ pbz(DistanceFromAfrica) + pbz(
NbSpeakers) + pbz(LgDensity) + random(Family))
model.gamlss.DEL.sigma <- update(model.gamlss.DEL.mu, ~ pbz(DistanceFromAfrica) + pbz(
NbSpeakers) + pbz(LgDensity) + random(Family), what="sigma")
model.gamlss.DEL.nu <- update(model.gamlss.DEL.sigma, ~ pbz(DistanceFromAfrica) + pbz(
NbSpeakers) + pbz(LgDensity) + random(Family), what="nu")

# SICHEL
model.gamlss.SICHEL.empty <- gamlss(Segments ~ 1, data = lg.df, family=SICHEL(mu.link=
"identity"), mu.start = 38,
                                 control = gamlss.control(n.cyc = 200), i.control=
                                 glim.control(bf.cyc = 200))
model.gamlss.SICHEL.mu <- update(model.gamlss.SICHEL.empty, ~ pbz(DistanceFromAfrica) + pbz(
NbSpeakers) + pbz(LgDensity) + random(Family))
model.gamlss.SICHEL.sigma <- update(model.gamlss.SICHEL.mu, ~ pbz(DistanceFromAfrica) + pbz(
NbSpeakers) + pbz(LgDensity) + random(Family), what="sigma")
model.gamlss.SICHEL.nu <- update(model.gamlss.SICHEL.sigma, ~ pbz(DistanceFromAfrica) + pbz(
NbSpeakers) + pbz(LgDensity) + random(Family), what="nu")

# BCCG
model.gamlss.BCCG.empty <- gamlss(Segments ~ 1, data = lg.df, family=BCCG(mu.link="identity")
```

```
, mu.start = 38,
                                   control = gamlss.control(n.cyc = 200), i.control=
                                   glim.control(bf.cyc = 200))
model.gamlss.BCCG.mu <- update(model.gamlss.BCCG.empty, ~ pbz(DistanceFromAfrica) + pbz(
NbSpeakers) + pbz(LgDensity) + random(Family))
model.gamlss.BCCG.sigma <- update(model.gamlss.BCCG.mu, ~ pbz(DistanceFromAfrica) + pbz(
NbSpeakers) + pbz(LgDensity) + random(Family), what="sigma")
model.gamlss.BCCG.nu <- update(model.gamlss.BCCG.sigma, ~ pbz(DistanceFromAfrica) + pbz(
NbSpeakers) + pbz(LgDensity) + random(Family), what="nu")

# BCT
model.gamlss.BCT.empty <- gamlss(Segments ~ 1, data = lg.df, family=BCT(mu.link="identity"),
mu.start = 38,
                                   control = gamlss.control(n.cyc = 200), i.control=
                                   glim.control(bf.cyc = 200))
model.gamlss.BCT.mu <- update(model.gamlss.BCT.empty, ~ pbz(DistanceFromAfrica) + pbz(
NbSpeakers) + pbz(LgDensity) + random(Family))
model.gamlss.BCT.sigma <- update(model.gamlss.BCT.mu, ~ pbz(DistanceFromAfrica) + pbz(
NbSpeakers) + pbz(LgDensity) + random(Family), what="sigma")
model.gamlss.BCT.nu <- update(model.gamlss.BCT.sigma, ~ pbz(DistanceFromAfrica) + pbz(
NbSpeakers) + pbz(LgDensity) + random(Family), what="nu")
model.gamlss.BCT.tau <- update(model.gamlss.BCT.nu, ~ pbz(DistanceFromAfrica) + pbz(
NbSpeakers) + pbz(LgDensity) + random(Family), what="tau")


# °°°°°°°°°°°°°°°°°°°°°°°°°°°°°°°°°°°°°°°°°°°°°°°°°°°°
# GAMLSS, Investigating deviance, AIC, BIC and df ####
# °°°°°°°°°°°°°°°°°°°°°°°°°°°°°°°°°°°°°°°°°°°°°°°°°°°°

models <- list(model.gamlss.IG.mu, model.gamlss.IG.sigma,
               model.gamlss.PIG.mu, model.gamlss.PIG.sigma,
               model.gamlss.DEL.mu, model.gamlss.DEL.sigma, model.gamlss.DEL.nu,
               model.gamlss.BCCG.mu, model.gamlss.BCCG.sigma, model.gamlss.BCCG.nu,
               model.gamlss.BCT.mu, model.gamlss.BCT.sigma, model.gamlss.BCT.nu,
               model.gamlss.BCT.tau
              )

myNames <- c("IG mu", "IG mu & sigma",
             "PIG mu", "PIG mu & sigma",
             "DEL mu", "DEL mu & sigma", "DEL mu, sigma & nu",
             "BCCG mu", "BCCG mu & sigma", "BCCG mu, sigma & nu",
             "BCT mu", "BCT mu & sigma", "BCT mu, sigma & nu", "BCT mu, sigma, nu & tau")

v <- lapply(models, get.results.gamlss)
df <- as.data.frame(data.table::transpose((v)))
colnames(df) <- c("Global deviance", "df for mu", "df for sigma", "df for nu", "df for tau",
"df", "AIC", "BIC")
rownames(df) <- myNames
zapsmall(df, digits=6)

# Checking the distribution of residuals with worm plots
wp(model.gamlss.DEL.nu, y.lim = 1.5)
wp(model.gamlss.BCT.sigma, y.lim = 1.5)
wp(model.gamlss.BCT.nu, y.lim = 1.5)
wp(model.gamlss.BCCG.nu, y.lim = 1.5)

# Residuals are much better with the BCCG and BCT distributions...
```

```
# °°°°°°°°°°°°°°°°°°°°°°°°°°°°°°°°°°°
# GAMLSS, Focusing on BCT GAMLSS ####
# °°°°°°°°°°°°°°°°°°°°°°°°°°°°°°°°°°°

summary(model.gamlss.BCT.nu)

# Getting details about the smooth terms
getSmo(model.gamlss.BCT.nu, "mu", which = 1)
getSmo(model.gamlss.BCT.nu, "mu", which = 2)
getSmo(model.gamlss.BCT.nu, "mu", which = 3)
getSmo(model.gamlss.BCT.nu, "mu", which = 4)

getSmo(model.gamlss.BCT.nu, "sigma", which = 1)
getSmo(model.gamlss.BCT.nu, "sigma", which = 2)
getSmo(model.gamlss.BCT.nu, "sigma", which = 3)
getSmo(model.gamlss.BCT.nu, "sigma", which = 4)

getSmo(model.gamlss.BCT.nu, "nu", which = 1)
getSmo(model.gamlss.BCT.nu, "nu", which = 2)
getSmo(model.gamlss.BCT.nu, "nu", which = 3)
getSmo(model.gamlss.BCT.nu, "nu", which = 4)

getSmo(model.gamlss.BCT.tau, "tau", which = 1)
getSmo(model.gamlss.BCT.tau, "tau", which = 2)
getSmo(model.gamlss.BCT.tau, "tau", which = 3)
getSmo(model.gamlss.BCT.tau, "tau", which = 4)

# Computing a simplified model equivalent to model.gamlss.BCT.nu
model.gamlss.BCT.nu.reduced <- gamlss(Segments ~ pb(DistanceFromAfrica) + NbSpeakers +
LgDensity + random(Family),
                                      sigma.fo = ~ DistanceFromAfrica + NbSpeakers +
                                      LgDensity + random(Family),
                                      nu.fo = ~ DistanceFromAfrica + pb(NbSpeakers) +
                                      LgDensity,
                                      data = lg.df, family=BCT(mu.link="identity"), mu.start
                                      = 38,
                                      control = gamlss.control(n.cyc = 200), i.control=
                                      glim.control(bf.cyc = 200))

# Checking that the initial and reduced models are identical
summary(model.gamlss.BCT.nu.reduced)
summary(model.gamlss.BCT.nu)

deviance(model.gamlss.BCT.nu)
deviance(model.gamlss.BCT.nu.reduced)

AIC(model.gamlss.BCT.nu, model.gamlss.BCT.nu.reduced)

getSmo(model.gamlss.BCT.nu.reduced, "mu", which = 1)
getSmo(model.gamlss.BCT.nu.reduced, "mu", which = 2)
getSmo(model.gamlss.BCT.nu.reduced, "sigma", which = 1)
getSmo(model.gamlss.BCT.nu.reduced, "nu", which = 1)


# °°°°°°°°°°°°°°°°°°°°°°°°°°°°°°°°°°°°°°°°°°°°°°°°°°°°°
# GAMLSS, Assessing significance of the predictors ####
```

```
# °°°°°°°°°°°°°°°°°°°°°°°°°°°°°°°°°°°°°°°°°°°°°°°°°°°°°°°°°°°°°°°°

# Extracting the effective degrees of freedom of the various smooth terms to fix them

(edf.dist.mu <- getSmo(model.gamlss.BCT.nu.reduced, "mu", which = 1)$edf)
(edf.fam.mu <- getSmo(model.gamlss.BCT.nu.reduced, "mu", which = 2)$edf)
(edf.fam.sigma <- getSmo(model.gamlss.BCT.nu.reduced, "sigma", which = 1)$edf)
(edf.nbspk.nu <- getSmo(model.gamlss.BCT.nu.reduced, "nu", which = 1)$edf)

# Computing the model with fixed degrees of freedom for smooth terms
model.gamlss.BCT.nu.reduced.fx <- gamlss(Segments ~ pb(DistanceFromAfrica, df = edf.dist.mu-2
) + NbSpeakers + LgDensity + random(Family, df = edf.fam.mu),
                                         sigma.fo = ~ DistanceFromAfrica + NbSpeakers + 
                                         LgDensity + random(Family, df = edf.fam.sigma),
                                         nu.fo = ~ DistanceFromAfrica + pb(NbSpeakers, df = 
                                         edf.nbspk.nu-2) + LgDensity,
                                         data = lg.df, family=BCT(mu.link="identity"), 
                                         mu.start = 38,
                                        control = gamlss.control(n.cyc = 200), i.control=
                                        glim.control(bf.cyc = 200))

# Checking that smooth terms are ok
getSmo(model.gamlss.BCT.nu.reduced.fx, "mu", which = 1)
getSmo(model.gamlss.BCT.nu.reduced.fx, "mu", which = 2)
getSmo(model.gamlss.BCT.nu.reduced.fx, "sigma", which = 1)
getSmo(model.gamlss.BCT.nu.reduced.fx, "nu", which = 1)

summary(model.gamlss.BCT.nu.reduced.fx)

# Computing LR tests to assess significance of the various additive terms
dropterm(model.gamlss.BCT.nu.reduced.fx, what = "mu", test="Chisq")
dropterm(model.gamlss.BCT.nu.reduced.fx, what = "sigma", test="Chisq")
dropterm(model.gamlss.BCT.nu.reduced.fx, what = "nu", test="Chisq")



# °°°°°°°°°°°°°°°°°°°°°°°°°°°°°°°°
# GAMLSS, Graphical Outputs ####
# °°°°°°°°°°°°°°°°°°°°°°°°°°°°°°°°

(p1 <- get.figure.partial.component.gamlss(model.gamlss.BCT.nu.reduced, lg.df, 
"DistanceFromAfrica", 1, "Smooth term for Distance From Africa", "s(Distance from Africa)"))
(p2 <- get.figure.partial.component.gamlss(model.gamlss.BCT.nu.reduced, lg.df, "NbSpeakers", 
2, "Number of speakers", "Number of Speakers"))
(p3 <- get.figure.partial.component.gamlss(model.gamlss.BCT.nu.reduced, lg.df, "LgDensity", 3
, "Local linguistic density", "Local linguistic density"))

(q1 <- get.figure.partial.component.gamlss(model.gamlss.BCT.nu.reduced, lg.df, 
"DistanceFromAfrica", 1, "Distance From Africa", "Distance from Africa", "sigma"))
(q2 <- get.figure.partial.component.gamlss(model.gamlss.BCT.nu.reduced, lg.df, "NbSpeakers", 
2, "Number of speakers", "Number of Speakers", "sigma"))
(q3 <- get.figure.partial.component.gamlss(model.gamlss.BCT.nu.reduced, lg.df, "LgDensity", 3
, "Local linguistic density", "Local linguistic density", "sigma"))

merged.figures <- arrangeGrob(p1, p2, p3, q1, q2, q3, nrow=2, ncol=3)
plot(merged.figures)

ggsave("GAMLSS_BCT_NU_SMOOTHERS_MU_SIGMA.tiff", plot = merged.figures, device = 'tiff', path 
```

```
= folder,
      scale = 1, width = 85*3.125, height = 85*3.125*2/3, units = "mm", dpi = 300,
      compression = "lzw")


merged.figures <- arrangeGrob(get.figure.expected.values.vs.residuals(model.gamlss.IG.mu,
"(IG, \U03bc)"),
                              get.figure.qq.plot(residuals(model.gamlss.IG.mu), "(IG,
                              \U03bc)"),
                              get.figure.expected.values.vs.residuals(model.gamlss.DEL.nu,
                              "(DEL, \U03bc, \U03c3 & \U03bd)"),
                              get.figure.qq.plot(residuals(model.gamlss.DEL.nu), "(DEL,
                              \U03bc, \U03c3 & \U03bd)"),
                              get.figure.expected.values.vs.residuals(model.gamlss.BCT.mu,
                              "(BCT, \U03bc, \U03c3 & \U03bd)"),
                              get.figure.qq.plot(residuals(model.gamlss.BCT.mu), "(BCT,
                              \U03bc, \U03c3 & \U03bd)"),
                              nrow=3, ncol=2)
plot(merged.figures)
ggsave("GAMLSS_COMPARISON_RESIDUALS.tiff", plot = merged.figures, device = 'tiff', path =
folder,
      scale = 1, width = 85*3.125, height = 85*3.125*2/3, units = "mm", dpi = 300,
      compression = "lzw")


tiff(file = paste0(folder, "GAMLSS_WP.tiff"), width = 3200, height = 1565, units = "px",
compression = "lzw", res = 300)
par(mfrow=c(1,3))
wp(model.gamlss.IG.mu, ylim.all=1.5)
title(main="(IG, \U03bc)")
wp(model.gamlss.DEL.nu, ylim.all=1.5)
title(main="(DEL, \U03bc, \U03c3 & \U03bd)")
wp(model.gamlss.BCT.nu, ylim.all=1.5)
title(main="(BCT, \U03bc, \U03c3 & \U03bd)")
par(mfrow=c(1,1))
dev.off()

# Note: the Unicode characters are for the greek letters for mu, sigma and nu
```
